# Assessing Knowledge, Attitudes, and Practices Towards Herpes Zoster and Vaccination in Japan Using the Capability-Opportunity-Motivation-Behavior Model: a Mixed-Methods Study

**DOI:** 10.3389/ijph.2025.1608121

**Published:** 2026-03-13

**Authors:** Yuki Suzaki, Shinichi Imafuku, Jing Chen, Jennifer Si, Viola Xiang, Vince Grillo, Takahiko Imai, Jerusha Naidoo, Sumitra Shantakumar

**Affiliations:** 1 GSK, Tokyo, Japan; 2 Department of Dermatology, Faculty of Medicine, Fukuoka University, Fukuoka, Japan; 3 GSK, Singapore, Singapore; 4 Oracle Life Sciences, Singapore, Singapore

**Keywords:** herpes zoster, herpes zoster vaccination, Japan, knowledge attitudes and practices, survey

## Abstract

**Objectives:**

To assess knowledge, attitudes, and practices (KAP) towards herpes zoster (HZ) and HZ vaccination in Japan.

**Methods:**

This mixed-methods study was conducted across two phases. In Phase 1, concept elicitation interviews were conducted with the public (N = 24) and physicians (N = 6), and the Capability-Opportunity-Motivation-Behavior model of behavioral change was used to identify themes surrounding KAP. These themes were validated in Phase 2 via self-administered quantitative surveys conducted with a larger group of respondents (public: N = 600; physicians: N = 60).

**Results:**

Despite high awareness of HZ (92.9%–94.0%) and HZ vaccination (76.0%–80.4%) among the public, knowledge about HZ, HZ vaccination rates (13.1%–32.0%), and intention to vaccinate (12.6%–18.2% among non-HZ-vaccinated respondents) were low. Public respondents were likely to vaccinate against HZ upon physician recommendation (78.7%–84.0%), but physician recommendation was limited by barriers including perceived low patient willingness (51.7%) and vaccine cost (51.7%). Various forms of government support could encourage patient-physician discussions regarding HZ and aid HZ vaccination uptake among the public (30.0%–53.3%).

**Conclusion:**

These findings may inform public health strategies to overcome barriers to HZ vaccine uptake in Japan.

## Introduction

Herpes zoster (HZ) is a viral disease caused by reactivation of latent varicella zoster virus in the dorsal root ganglia [1, 2]. Characterized by a painful dermatomal rash [1, 2], HZ can cause debilitating long-term complications including postherpetic neuralgia (PHN; i.e., pain persisting for >90 days post-rash onset), vision loss, and long-lasting motor deficits [3, 4]. HZ has substantial impact on quality-of-life (QoL), including physical, social, and emotional functioning [4, 5].

The risk of developing HZ increases with age, especially after 50 years, due to age-related decline in immunity [4]. Certain comorbidities with increased prevalence in older adults, including diabetes and cardiovascular diseases [6, 7], have also been associated with elevated HZ risk [8–10]. There is thus concern about HZ burden increasing in ageing populations such as Japan [11], where HZ incidence is highest in adults aged ≥50 years [1].

While HZ is generally treated with antiviral drugs and analgesics [12], these have demonstrated limited efficacy against PHN and minimal impact on QoL [4, 13–15]. Conversely, HZ vaccination (a proactive approach to HZ prevention) may reduce HZ risk and mitigate the severity and impact of HZ symptoms [16–18]. Two HZ vaccines are currently available in Japan: freeze-dried live attenuated varicella vaccine [19, 20] and recombinant zoster vaccine (RZV) [21–23]. Both are approved for HZ prevention in adults aged ≥50 years, and RZV additionally in adults aged ≥18 years at increased HZ risk.

HZ vaccination was recently added to Japan’s National Immunization Program (NIP) for adults aged 65 years and those aged 60–64 years with weakened immunity due to human immunodeficiency virus (HIV) infection [24]. However, Japan’s long-standing history of vaccine hesitancy may hinder vaccine uptake; with major safety concerns raised by Japanese citizens and health ministry regarding a range of vaccines in the past, vaccine confidence in Japan is one of the lowest worldwide [25, 26].

To date, limited studies have described knowledge, attitudes, and practices (KAP) towards HZ and HZ vaccination among adults aged ≥50 years in Japan [15], suggesting an under-recognition of HZ burden and the importance of HZ prevention. Alongside factors influencing HZ vaccination at an individual level, effective communications with healthcare professionals (HCPs) are also crucial in improving public knowledge about health issues and influencing vaccine-seeking behaviors [27, 28]. A useful framework for identifying drivers of HZ vaccination is the Capability-Opportunity-Motivation-Behavior (COM-B) model of behavioral change (Supplementary Figure S1). Commonly used to design and evaluate behavioral change interventions and policies, the model posits that behavioral change arises from targeting the interinfluencing components of capability, opportunity, and motivation [29].

This study therefore sought to use the COM-B model to assess KAP towards HZ and HZ vaccination among the public and physicians in Japan, and explore differences in KAP across different respondent subgroups; these insights may inform local public health interventions surrounding HZ prevention. This is an extension of a previous Asia-Pacific regional study involving Hong Kong, Republic of Korea, Singapore, and Taiwan, which identified knowledge gaps and cognitive biases that could be addressed to improve HZ-related practices in the region [30].

## Methods

### Study Design

A mixed-methods study was conducted across two phases with Japanese respondents (Supplementary Figure S2). In Phase 1, exploratory, concept elicitation, one-to-one virtual interviews (∼45 min) were conducted in Japanese with the public and physicians in January–March 2023. An interview discussion guide was developed based on a literature review of KAP regarding HZ and HZ vaccination, and discussions with a local expert engaged to provide expert opinions on study material. Relevant themes surrounding KAP related to HZ and HZ vaccination were summarized from the interview responses, using the COM-B model to identify behavioral gaps or barriers [29]. Further details on the Phase 1 study design and data analysis were previously published [30].

The themes identified in Phase 1 were quantitatively validated in Phase 2, which involved a self-administered cross-sectional survey (∼30 min) conducted with a larger group of respondents in April–May 2023. The survey questionnaire was designed based on themes elicited in Phase 1, which were categorized into COM-B domains centered around topics including knowledge, motivations, and opportunity, capturing attitudes and behaviors towards HZ and HZ vaccines (Supplementary Appendix S1, S2); the literature review and expert input were also considered. The questionnaire was then translated into Japanese and completed by all respondents online. Descriptive analyses of all questions were conducted according to respondent groups and subgroups, and presented as counts, percentages, means, and standard deviations depending on the scale of the item/measure. Bivariate comparisons between different respondent groups and subgroups were also conducted (Pearson’s Chi Square tests and one-way analyses of variance [ANOVA] for categorical and continuous variables, respectively).

A protocol amendment to the prior approved regional study was submitted to the above-country central Institutional Review Board (IRB), Pearl IRB, to include Japan as a participating country; exemption from full review was obtained (#21-CERN-104). A statement of informed consent was provided to all potential respondents who met eligibility criteria. Informed consent was obtained electronically for both phases.

### Study Population

The study was conducted with public (i.e., non-physician) and physician respondents in Japan. Based on previously published mixed-methods studies (not HZ-related) [31–33], a sample size of 24–30 public respondents in Phase 1 was considered sufficient to identify significant themes for validation in Phase 2. Similarly, based on previously published studies in HZ [34–36], a sample size of 450–600 public respondents in Phase 2 was recommended; sample sizes of public respondent subgroups and physicians were estimated based on feasibility of recruitment.

In both phases, public respondents were recruited via Kantar Profiles panel and/or its partners’ databases through purposive sampling. Potential respondents were screened for eligibility, via phone calls/emails (Phase 1) or an online screener (Phase 2), based on the prespecified inclusion and exclusion criteria (Supplementary Table S1). Subgroups of public respondents were recruited: (i) HZ-naïve adults aged ≥50 years, (ii) adults aged ≥50 years, vaccinated with zoster vaccine live or RZV, (iii) current or former HZ patients aged ≥50 years, and (iv) working/financially independent adults aged 30–49 years, with parents aged ≥50 years. The (iv) group was included due to the important role children often play in healthcare decision-making and financial support for their elderly parents in Asian societies [37].

Physician respondents were recruited through purposive sampling via HCP databases consolidated from hospital websites and public HCP registries in Japan. General practitioners (GPs), pain clinicians, and dermatologists were recruited, based on the prespecified eligibility criteria (Supplementary Table S1).

Respondents who participated in the initial concept elicitation interviews (Phase 1) were excluded from the online quantitative surveys (Phase 2).

## Results

### Phase 1 (Concept Elicitation)

#### Demographics

A total of 24 members of the public and 6 physicians were interviewed. Public respondents included 6 non-HZ-vaccinated adults aged ≥50 years, 6 HZ-vaccinated adults aged ≥50 years, 6 current or former HZ patients aged ≥50 years, and 6 working/financially independent adults aged 30–49 years with parents aged ≥50 years (hereafter described as “adult children”). Of all adults aged ≥50 years (hereafter described as “older adults”), half were aged ≥65 years. Physician respondents included 2 GPs, 2 pain clinicians, and 2 dermatologists.

#### The Public

Public respondents reported some awareness of HZ based on experiences shared by HZ-experienced family/friends and media coverage. Current knowledge of HZ symptoms, risk factors, long-term complications, and treatment options was limited; at least 1 member of each group had misconceptions about HZ.

Most respondents were unfamiliar with HZ vaccines, and knowledge was limited even among HZ patients. Nevertheless, respondents expressed desire to learn more about HZ and HZ vaccination, including the risks and benefits of, suitable target population for, and ways to access HZ vaccines. Physicians and the government were perceived as reliable sources of information with significant influence on their vaccination decisions. Respondents also shared that personal stories from HZ-experienced family/friends influenced their attitudes towards HZ and HZ vaccines, which in turn affected vaccination behavior.

Across all respondent groups, avoiding pain and long-term complications of HZ were reported as key drivers to seeking HZ vaccination. Respondents were however deterred by the high cost of HZ vaccination, with adult children and HZ-vaccinated individuals reporting government subsidy as a motivation to receive vaccination.

#### Physicians

Physicians demonstrated ample knowledge of HZ, its symptoms and long-term complications, the at-risk population, and its risk of recurrence. They could also assess different HZ vaccines by factors including their nature, contraindications, side effects, and costs. Physicians reportedly prioritized vaccination for diseases with greater prevalence and perceived severity (e.g., pneumococcal disease, influenza), as well as on patient’s request. Concerns regarding out-of-pocket vaccine expense for patients and perceived hesitancy/unwillingness among patients to get vaccinated were additional barriers to their active recommendation of HZ vaccination. They acknowledged the need for government support, through official government information and subsidies, for clinical decision-making regarding HZ.

### Phase 2 (Quantitative Validation): The Public

#### Demographics

After screening out respondents who were either unaware of HZ or had rejected preventive vaccines (Supplementary Figure S3), 550 older adults and 50 adult children were recruited (Table 1). Among the older adults included in the study, 13.1% had been vaccinated against HZ and 36.4% were current or former HZ patients. Majority were aged ≥60 years (56.7%) and mainly responsible for making their own decisions (69.3%). Among the 50 adult children included in the study, 92.0% had parents aged >60 years and 60.0% had family history of HZ; 32.0% had parents who were vaccinated against HZ, and 42.0% had parents with a current or former HZ diagnosis. More than half (54.0%) of adult children were mainly responsible for decision-making for their parent(s). Older adult and adult children respondents were recruited uniformly across regions in Japan.

**TABLE 1:** Demographics and characteristics of public respondents (older adults and adult children) (Japan, 2023).

Demographic/characteristic	Older adults										Adult children	
	Overall (N = 550)		HZ vaccination status				HZ history				Overall (N = 50)	
			Vaccinated (N = 72)		Non-vaccinated (N = 478)		HZ patients (N = 200)		HZ naïve (N = 350)			
	n	%	n	%	n	%	n	%	n	%	n	%
Gender												
Male	318	57.8	38	52.8	280	58.6	125	62.5	193	55.1	27	54.0
Female	232	42.2	34	47.2	198	41.4	75	37.5	157	44.9	23	46.0
Age												
30–35 years	N/A		N/A		N/A		N/A		N/A		10	20.0
36–40 years	N/A		N/A		N/A		N/A		N/A		12	24.0
41–44 years	N/A		N/A		N/A		N/A		N/A		13	26.0
45–49 years	N/A		N/A		N/A		N/A		N/A		15	30.0
50–55 years	126	22.9	15	20.8	111	23.2	42	21.0	84	24.0	N/A	
56–60 years	112	20.4	16	22.2	96	20.1	45	22.5	67	19.1	N/A	
61–64 years	94	17.1	13	18.1	81	16.9	35	17.5	59	16.9	N/A	
≥65 years	218	39.6	28	38.9	190	39.7	78	39.0	140	40.0	N/A	
Parent’s age												
50–54 years	N/A		N/A		N/A		N/A		N/A		0	0
55–59 years	N/A		N/A		N/A		N/A		N/A		4	8.0
60–64 years	N/A		N/A		N/A		N/A		N/A		8	16.0
≥65 years	N/A		N/A		N/A		N/A		N/A		38	76.0
Education level												
Elementary or high school	115	20.9	14	19.4	101	21.1	39	19.5	76	21.7	7	14.0
Vocational school	84	15.3	11	15.3	73	15.3	24	12.0	60	17.1	7	14.0
University and above	348	63.3	47	65.3	301	63.0	136	68.0	212	60.6	36	72.0
Prefer not to answer	3	0.5	0	0	3	0.6	1	0.5	2	0.6	0	0
Employment status												
Employed	N/A		N/A		N/A		N/A		N/A		46	92.0
Not employed but financially independent	N/A		N/A		N/A		N/A		N/A		4	8.0
Living with parent aged ≥50 years												
Yes	N/A		N/A		N/A		N/A		N/A		28	56.0
No	N/A		N/A		N/A		N/A		N/A		22	44.0
HZ vaccination status											Parent’s HZ vaccination status	
Yes	72	13.1	72	100.0	0	0	22	11.0	50	14.3	16	32.0
No	478	86.9	0	0	478	100.0	178	89.0	300	85.7	34	68.0
HZ diagnosis status											Parent’s HZ diagnosis status	
Former HZ diagnosis (recovered)	190	34.5	20	27.8	170	35.6	190	95.0	0	0	15	30.0
Current ongoing HZ	10	1.8	2	2.8	8	1.7	10	5.0	0	0	6	12.0
Does not have HZ	350	63.6	50	69.4	300	62.8	0	0.0	350	100.0	29	58.0
HZ disease severity												
*Valid n* ^a^	200		22		178		200		0		N/A	
Mild	100	50.0	4	18.2	96	53.9	100	50.0	-	-	N/A	
Moderate	92	46.0	16	72.7	76	42.7	92	46.0	-	-	N/A	
Severe	8	4.0	2	9.1	6	3.4	8	4.0	-	-	N/A	
Family history of HZ												
Yes	165	30.0	25	34.7	140	29.3	66	33.0	99	28.3	30	60.0
No	385	70.0	47	65.3	338	70.7	134	67.0	251	71.7	20	40.0
Role in decision-making											Role in decision-making for parent	
I am mainly responsible	381	69.3	61	84.7	320	66.9	145	72.5	236	67.4	27	54.0
We make decisions as a family unit	168	30.5	11	15.3	157	32.8	54	27.0	114	32.6	23	46.0
My children decide for me	1	0.2	0	0	1	0.2	1	0.5	0	0	N/A	

Older adult (aged ≥50 years) and adult children (aged 30–49 years, with parents aged ≥50 years) respondents included in the study were aware of HZ, and open to preventive vaccination. Data were rounded to the first decimal place and the sum of values may not total to 100%.

^a^ Disease severity was assessed among current/former older adult patients with HZ, only. HZ: herpes zoster; N/A: not applicable.

#### KAP Related to HZ

**FIGURE 1:**
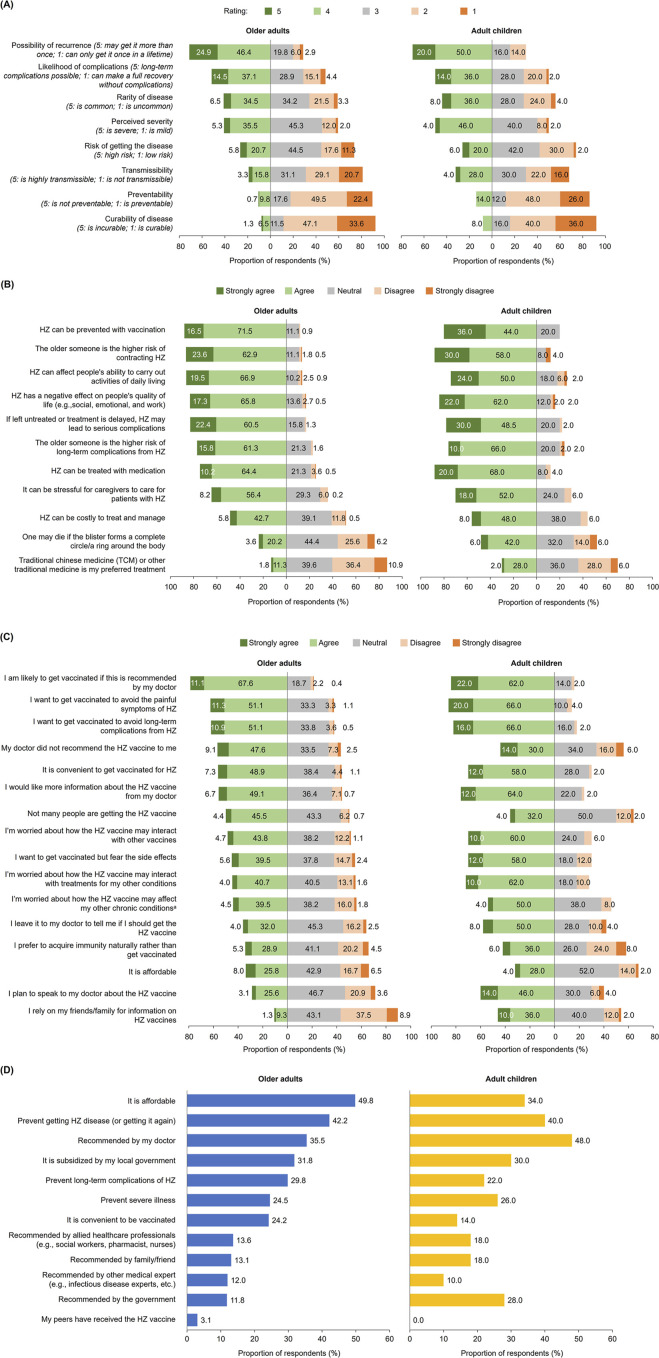
**(A)** Perceptions of herpes zoster, **(B)** attitudes towards herpes zoster, **(C)** attitudes towards herpes zoster vaccine, and **(D)** key drivers of herpes zoster vaccine, among the public (older adults aged ≥50 years and adult children aged 30–49 years with parents aged ≥50 years) (Japan, 2023). ^a^Examples include diabetes, hypertension and high cholesterol. Survey questions for public respondents (all statements surveyed are presented): **(A)** “Here are some statements others have said about HZ (shingles) as an illness. Please select a rating that is closest to how you feel about each statement (Japan, 2023).” **(B)** “Here are some other statements others have said about HZ. To what extent do you agree/disagree with these statements?” **(C)** “The following are statements about what people think and feel about HZ (shingles) vaccine. To what extent do you agree/disagree with these statements?” **(D)** “Which of the following factors are most important when deciding whether to take the HZ (shingles) vaccine for yourself/your elderly parents?” Data were rounded to the first decimal place and the sum of values may not total to 100%. HZ: herpes zoster.

All public respondents were aware of HZ based on symptomatic description of the disease using local terms. Some respondents (older adults: 7.1%; adult children: 6.0%) were not aware of the medical term for HZ. Most respondents (older adults: 71.3%; adult children: 70.0%) recognized that HZ may recur, and approximately half recognized that HZ may lead to long-term complications (51.6%; 50.0%) (Figure 1A). Among older adults and adult children, 26.5% and 26.0%, respectively, perceived themselves/their parents to be at high risk of developing HZ, while 28.9% and 32.0% perceived themselves/their parents at low risk. Most respondents acknowledged the potential negative impact of HZ on QoL (older adults: 83.1%; adult children: 84.0%) and agreed that caring for HZ patients can be stressful (64.5%; 70.0%) (Figure 1B). Significantly higher proportions of HZ-vaccinated versus non-HZ-vaccinated older adults understood that the risk of long-term complications increases with age (87.5% versus 75.5%, p = 0.0299), and that HZ negatively impacts QoL (91.7% versus 81.8%, p = 0.0411) (Supplementary Table S2).

Public respondents identified low immunity (older adults: 72.4%; adult children: 68.0%) and stress (50.4%; 48.0%) as the top 2 risk factors for developing HZ; less than half of respondents (35.5%–40.0%; 38.0%–40.0%) identified older age (≥50 or 65 years) as a risk factor (Supplementary Table S3). While most respondents recognized pain (older adults: 82.7%; adult children: 74.0%) and rash (74.7%; 76.0%) as symptoms of HZ, less than half recognized itchiness and dryness (42.2%; 44.0%) and skin numbness (30.9%; 30.0%) as symptoms. Long-term nerve pain (54.9%; 50.0%) and skin infection/scarring (45.8%; 56.0%) were the most common long-term complications of HZ identified. Consistently higher proportions of HZ-vaccinated than non-HZ-vaccinated older adults were able to identify the long-term complications of HZ, with knowledge of long-term complications such as loss of vision (p = 0.0003), loss of hearing (p = 0.0127), mood disorders (p = 0.0029), and facial nerve paralysis (p = 0.0040) significantly higher among HZ-vaccinated than non-HZ-vaccinated individuals (Supplementary Table S2).

Nearly all (97.0%) current or former HZ patients sought medical treatment at a clinic or hospital. Among 81 HZ patients who sought treatment at a primary care physician, around one-third (37.0%) delayed doing so (i.e., ≥4 days after rash onset), with majority of these patients rationalizing that they did not seek timely treatment due to their perception that symptoms were not severe (73.3%).

#### KAP Related to Vaccination

Majority of public respondents were aware of HZ vaccine(s) (older adults: 80.4%; adult children: 76.0%). Respondents most commonly indicated that HZ vaccination is required for patients with low immunity or weakened immune system (71.1%; 62.0%), no previous HZ vaccination (54.7%; 42.0%), and those aged >65 years (46.9%; 54.0%).

Most public respondents were likely to seek HZ vaccination for themselves/their parents if recommended by a doctor (older adults: 78.7%; adult children: 84.0%), and to avoid painful symptoms (62.4%; 86.0%) and long-term complications (62.0%; 82.0%) of HZ (Figure 1C). Significantly higher proportions of HZ-vaccinated versus non-HZ-vaccinated older adults were likely to seek HZ vaccination for themselves if recommended by a doctor (90.3% versus 77.0%, p = 0.0365), and to avoid painful symptoms (97.2% versus 57.1%, p < 0.0001) and long-term complications (95.8% versus 56.9%, p < 0.0001) of HZ (Supplementary Table S2). Less than half of older adults were concerned about how HZ vaccines may interact with treatments for other conditions (44.7%) or with other vaccines (48.5%), affect current comorbidities (44.0%), and cause side effects (45.1%); proportions were higher among adult children (72.0%, 70.0%, 54.0%, and 70.0%, respectively) (Figure 1C).

Among non-HZ-vaccinated older adults and adult children with non-HZ-vaccinated elderly parents, 12.6% and 18.2% intended to get vaccinated and get their parents vaccinated, respectively, while approximately half (older adults: 49.4%; adult children: 57.6%) were undecided. The top factors driving HZ vaccine uptake were vaccine affordability (older adults: 49.8%; adult children: 34.0%), prevention of HZ (42.2%; 40.0%), and physician recommendation (35.5%; 48.0%) (Figure 1D). Notably, 12.4% of older adults and 28.0% of adult children reported having received recommendation for HZ vaccination from physicians or other HCPs. Local government subsidies were also considered a key driver of HZ vaccine uptake in ∼3 of 10 respondents (older adults: 31.8%; adult children: 30.0%).

#### Information Sources and Influence

**FIGURE 2:**
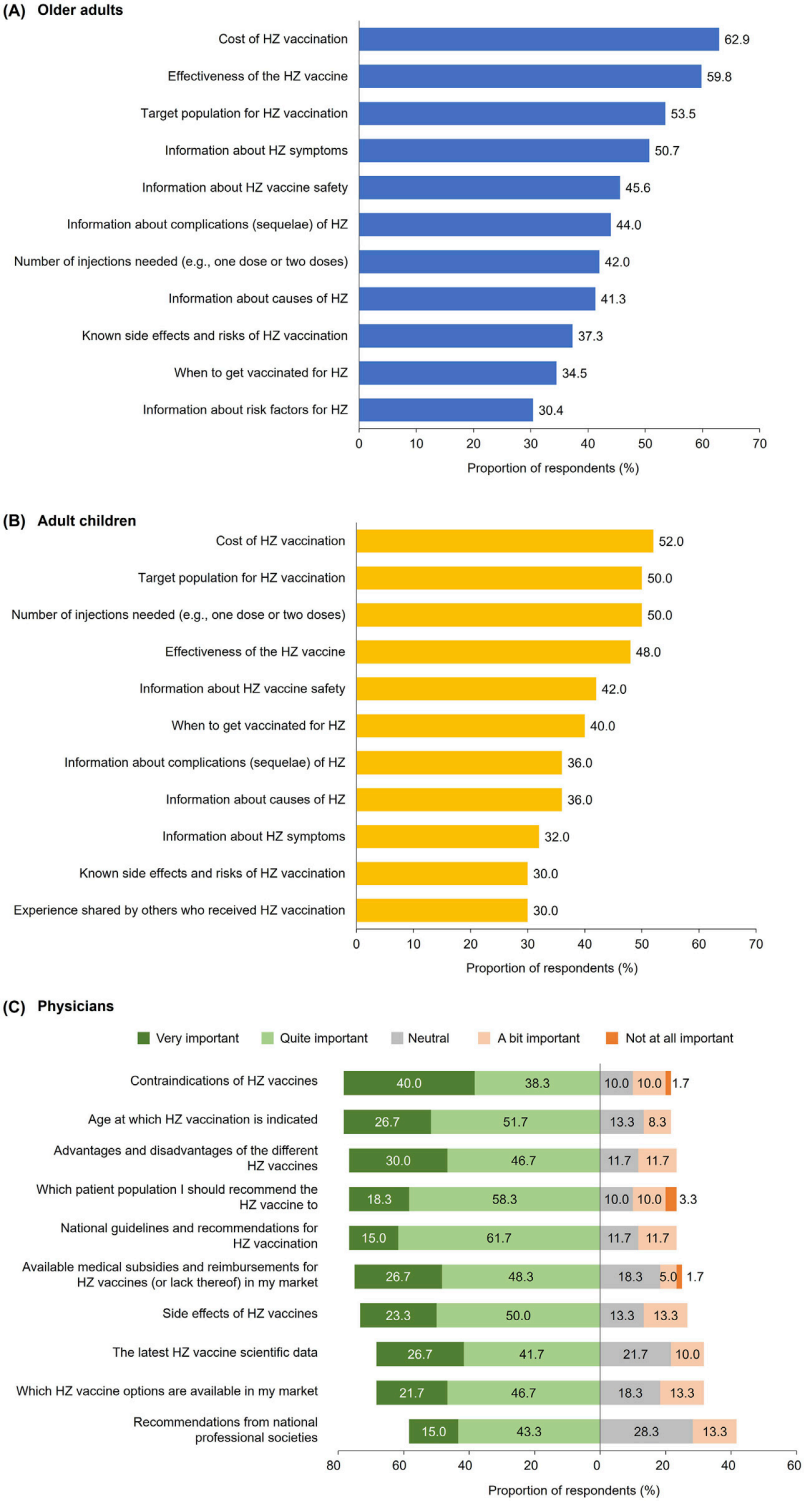
Herpes zoster and herpes zoster vaccination information topics of interest/importance among the public and physicians. **(A)** Topics of interest among older adults aged ≥50 years, **(B)** Topics of interest among adult children aged 30–49 years with parents aged ≥50 years, and **(C)** Topics of importance among physicians (Japan, 2023). **(A,B)** Survey question for public respondents: “What topics would you like to be provided with to help you learn more about HZ (shingles) disease and vaccination? Please select all that apply.” Statements surveyed that were selected by ≥30% of respondents are presented (Japan, 2023). **(C)** Survey question for physician respondents: “Here are some statements that other clinicians have told us are important to know about Herpes Zoster vaccine. Please rate the importance for you personally.” Percentages are the sum of respondents who indicated “Very important” and “Quite important” to each statement. Data were rounded to the first decimal place and the sum of values may not total to 100%. HZ: herpes zoster.

Local media was the most common source of information on HZ and HZ vaccination across all respondents (older adults: 40.0%–47.6%; adult children: 36.0%), while HCPs were the most trusted (79.1%; 70.0%) and preferred (72.2%; 60.0%) information source. Out of several HZ disease and vaccination-related topics, older adults were most interested in the cost (62.9%) or effectiveness of HZ vaccine (59.8%), and the target population for HZ vaccination (53.5%) (Figure 2A). The top topics of interest for adult children were similar: cost of HZ vaccine (52.0%), target population for HZ vaccination (50.0%), and number of injections needed (50.0%) (Figure 2B).

### Phase 2 (Quantitative Validation): Physicians

#### Demographics

A total of 60 physicians, comprising 20 GPs (33.3%), 20 pain clinicians (33.3%), and 20 dermatologists (33.3%), were recruited (Table 2). Most physicians (81.7%) had ≥16 years of clinical experience. Physicians were recruited uniformly across regions in Japan.

**TABLE 2:** Demographics and characteristics of physician respondents (Japan, 2023).

Demographic/characteristic	Overall (N = 60)	
	n	%
Gender		
Male	52	86.7
Female	8	13.3
Age		
<50 years	29	48.3
≥50 years	31	51.7
Specialty		
General practitioner	20	33.3
Dermatologist	20	33.3
Pain clinician	20	33.3
Years of experience		
<16 years	11	18.3
≥16 years	49	81.7
HZ patient load (per month)		
0–3	18	30.0
5–10	20	33.3
15–90	22	36.7
Hospital setting		
University hospital	11	18.3
National/public hospital	18	30.0
Others (e.g., clinic, private hospital, municipal hospital)	31	51.7
Vaccines administered or prescribed as part of routine clinical practice		
Influenza vaccine	46	76.7
Pneumococcal vaccine	43	71.7
COVID-19 vaccine	41	68.3
HZ vaccine	52	86.7

Data were rounded to the first decimal place and the sum of values may not total to 100%. COVID-19: coronavirus disease; HZ: herpes zoster.

#### KAP Related to HZ

Majority of physicians knew the risk factors for HZ, with over three-quarters identifying these as taking immunosuppressive medications (85.0%), being ≥50 years of age (78.3%), and having chronic medical conditions (76.7%) (Supplementary Table S4). While most physicians identified PHN (96.7%) and facial nerve paralysis (63.3%) as long-term complications associated with HZ, less than half recognized other long-term complications such as loss of vision (46.7%) and skin infection/scarring (46.7%).

Awareness and knowledge of the local HZ incidence rate were low among the included physicians; overall, 13.3% were unaware and 28.3% (GPs: 30.0%; pain clinicians: 25.0%; dermatologists: 30.0%) identified the incidence rate as 10 cases per 1,000 patient years, with similar proportions believing the incidence rate was higher or lower.

**FIGURE 3:**
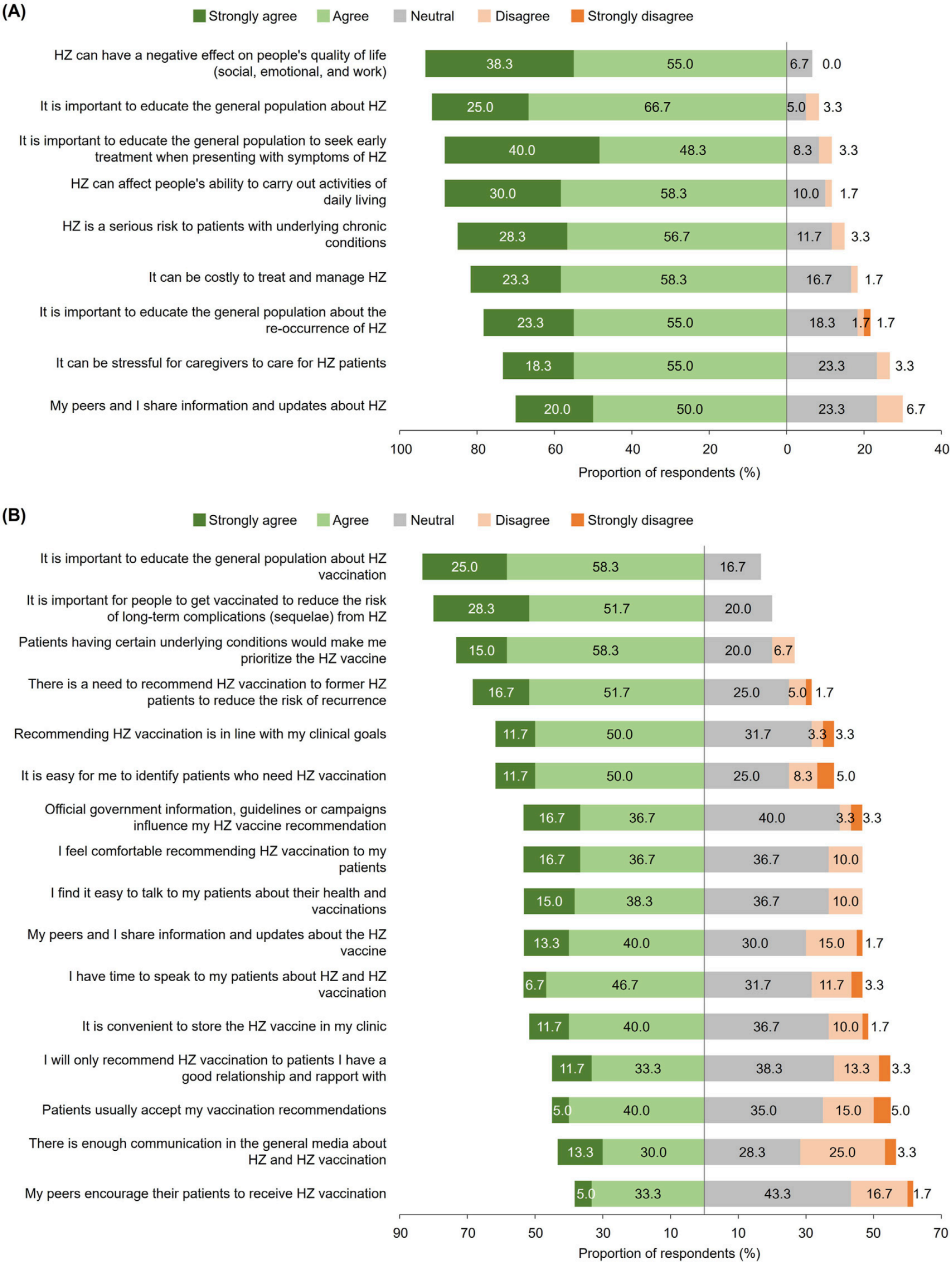
Perceived importance of **(A)** herpes zoster-related and **(B)** herpes zoster vaccine-related statements among physicians (Japan, 2023). Survey questions for physician respondents: **(A)** “Here are some statements about HZ disease. To what extent do you agree/disagree with the statements? Please answer using the following scale: Strongly agree, Agree, Neutral, Disagree, Strongly disagree.” **(B)** “Here are some statements about HZ vaccination. To what extent do you agree/disagree with the statements? Please answer using the following scale: Strongly agree, Agree, Neutral, Disagree, Strongly disagree.” Data are presented here for all physician respondents; all statements surveyed are presented. Data were rounded to the first decimal place and the sum of values may not total to 100%. HZ: herpes zoster.

Most physicians agreed that HZ negatively impacts overall QoL (93.3%), and that it is important to educate the public about both HZ disease (91.7%) and seeking treatment early on presentation of HZ symptoms (88.3%; Figure 3A).

#### KAP Related to Vaccination

Majority of physicians (85.0%) perceived HZ vaccination to be important to recommend to their patients aged ≥50 years, and 78.3% recommended, prescribed, and/or administered it to their patients in this age group within the 6-month period prior to the survey. Before recommending HZ vaccination to those aged ≥50 years, physicians most commonly considered the patient’s level of immunosuppression or severity of underlying diseases (70.0%), patient willingness or motivation to be vaccinated (68.3%), and age (66.7%).

The following HZ vaccine-related information topics were of greatest importance to physicians: contraindications to HZ vaccines (78.3%) and age at which HZ vaccination is indicated (78.3%) (Figure 2C). Over three-quarters of physicians (76.7%) deemed national guidelines and recommendations for HZ vaccines to be a topic of importance.

Majority agreed that it is important to educate the general population about HZ vaccination (83.3%), for people to be vaccinated to reduce risks of long-term complications from HZ (80.0%), and to prioritize patients with certain underlying conditions for HZ vaccination (73.3%) (Figure 3B). Notably, 53.3% of physicians agreed that official government information, and guidelines or campaigns would influence their recommendation of HZ vaccination.

#### Physician-Patient Communication

**FIGURE 4:**
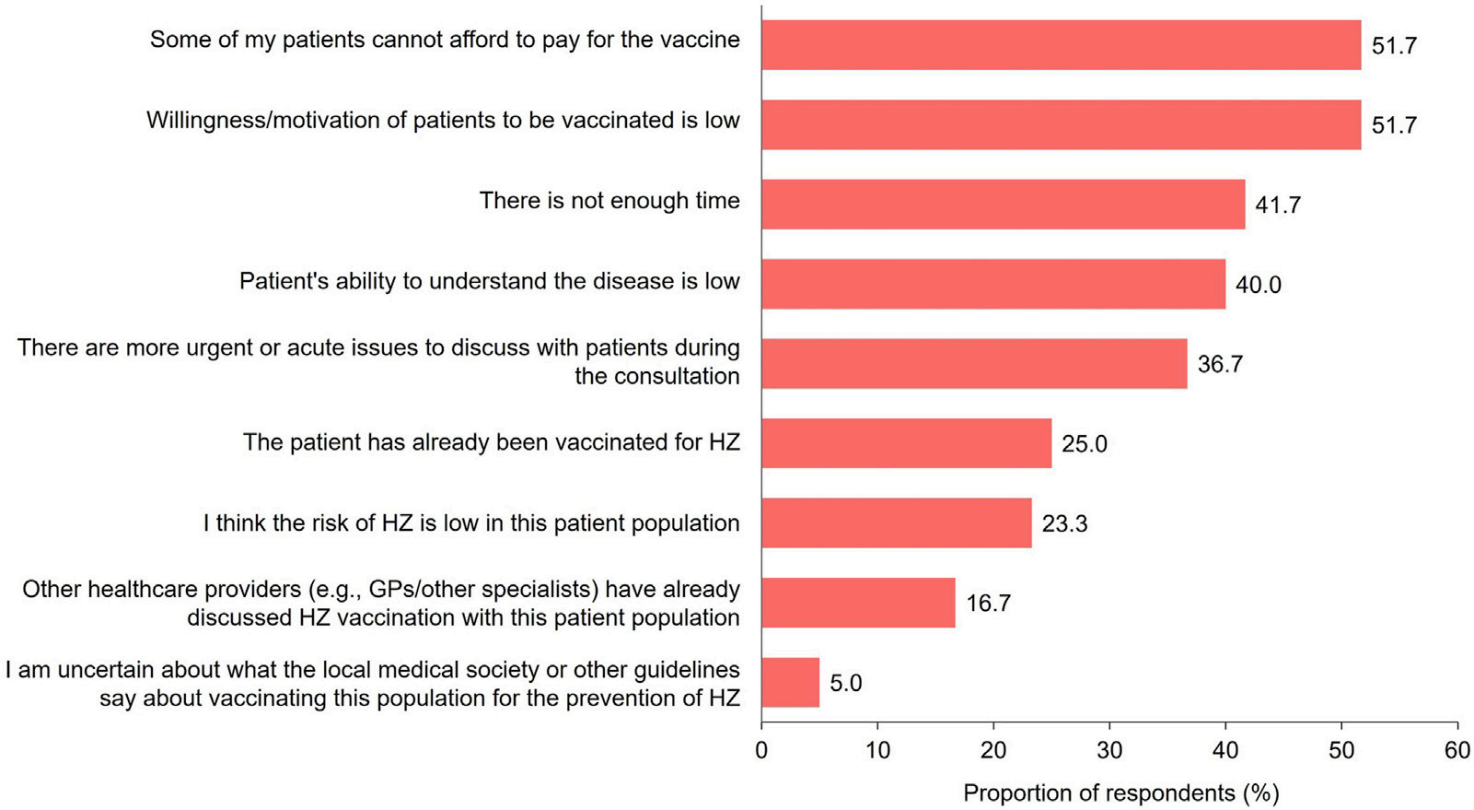
Perceived barriers among physicians when communicating with patients about herpes zoster and herpes zoster vaccination (Japan, 2023). Survey question for physician respondents: “Under what circumstances do you not initiate conversations with adult patients aged ≥50 years regarding HZ vaccination?” Data are presented here for all physician respondents; all options surveyed are presented. GP: general practitioner; HZ: herpes zoster.

Physicians recounted having initiated conversations about HZ disease and vaccination with a mean proportion of 25.3% patients aged ≥50 years, in the 1-year period prior to the survey. They identified the cost of vaccination (51.7%) and patient willingness to be vaccinated (51.7%) as the top 2 barriers when initiating conversations about HZ vaccination with patients aged ≥50 years (Figure 4). Other factors such as limited time during appointments (41.7%) and patient’s low ability to understand the disease (40.0%) were also perceived as barriers.

## Discussion

This study is among the first to evaluate KAP regarding HZ and HZ vaccination among the public and physicians in Japan. While study findings indicated high (>75%) awareness of HZ and HZ vaccines among the public, knowledge about HZ symptoms, long-term complications, risk, risk factors, and management was lacking, mirroring trends observed in other countries [35, 38, 39]. HZ-vaccinated individuals had greater awareness and knowledge of HZ risk factors and long-term complications, suggesting knowledge gaps could underlie reluctance to vaccinate against HZ. Studies have likewise shown that more non-HZ-vaccinated than HZ-vaccinated individuals perceived HZ could be “controlled” without a vaccine [39], and that greater HZ knowledge was associated with higher vaccine acceptability [40].

Despite high (>75%) awareness of the negative impact of HZ on QoL, only ∼1 in 10 older adults and 3 in 10 older adult parents of adult children were fully vaccinated against HZ. The intention to get vaccinated/get their parents vaccinated against HZ was also low among unvaccinated older adults/adult children with unvaccinated parents. This discordance between awareness of disease burden and vaccination practice could be attributed to barriers to HZ vaccine uptake such as lack of physician recommendation and vaccine affordability, both of which were identified as key drivers of HZ vaccination.

Physician recommendation was identified as a key factor influencing HZ vaccination-seeking behavior among the public; past studies have similarly shown that physician recommendation substantially increased acceptability of HZ vaccination, and even reversed patient rejection of HZ vaccination [36, 38–42]. Furthermore, the apparent congruence between rate of vaccine recommendation and vaccination status in Japan (66.7% of HZ-vaccinated older adults received HZ vaccine recommendation from family/friends/physicians/other HCPs, versus 15.5% of non-HZ-vaccinated older adults) suggests that recommendation could drive vaccine uptake. A previous study also found that HZ-vaccinated individuals were more likely than non-HZ-vaccinated individuals to have been recommended the HZ vaccine by vaccinated relatives/friends or HCPs [39].

However, physician-perceived barriers could limit conversations with patients regarding HZ and HZ vaccination. Notably, only one-quarter of physicians had initiated such conversations during their consultations in the 1-year period prior to the survey, with half potentially refraining from recommending HZ vaccines due to perceived low patient willingness/motivation to vaccinate and concerns about vaccine affordability. Correspondingly, a global systematic review and meta-analysis reported financial concerns as contributing factors to vaccination unwillingness, with only 1 in 2 individuals willing to vaccinate against HZ [42]. In the current study, the public’s low intent to vaccinate could also relate to their lack of knowledge about HZ long-term complications; only about half understood that HZ could cause long-term complications. Physician knowledge of long-term HZ complications was likewise lacking, with under half recognizing complications other than PHN and facial nerve paralysis. Given that preventing long-term complications of HZ would motivate the public’s vaccination-seeking behavior and is considered by physicians to be an important rationale for HZ vaccination, improving knowledge of the negative effects of HZ could encourage physicians to initiate HZ-related conversations with patients and increase patient willingness to seek vaccination.

Furthermore, vaccine affordability/cost was the top topic-of-interest and driving factor for vaccine uptake among the public. This is consistent with previous studies highlighting a lack of willingness to pay out-of-pocket for unsubsidized vaccines [35, 36, 38, 40, 43]. With nearly one-third of public respondents considering local government subsidy a key driver of HZ vaccine uptake, subsidies may support vaccination behavior among the community and HCPs locally. At the time of the study, HZ vaccination was not included in Japan’s NIP, and its recent integration into the NIP in April 2025 is anticipated to improve uptake by increasing access within the community. However, subsidies only partially cover vaccine cost, and are limited to adults aged ≥65 years and individuals aged 60–64 years immunocompromised due to HIV infection who are unable to perform activities of daily living, suggesting that financial barriers to HZ vaccination may remain.

Beyond the provision of subsidies, raising public awareness of their availability via government-led efforts and/or HCP sharing would be crucial in facilitating access. Official government information, guidelines, and campaigns related to HZ could inform physician recommendation of HZ vaccination and potentially address vaccine uptake. Moreover, public respondents indicated that local media was their most common source of information about HZ and HZ vaccination, while HCPs were their most trusted and preferred information source. Given that public trust in government sources, public health institutions, traditional news media outlets, and primary care physicians have been strong determinants of COVID-19 vaccine uptake in Japan [44, 45], these avenues may be effective means to reinforce best practices in HZ prevention and management.

Compared with the regional study (unpublished manuscript), KAP related to HZ and its vaccination in Japan differed to varying degrees. For example, vaccine affordability was a greater barrier to initiating HZ and HZ vaccination-related conversations for physicians in Japan (51.7%) than for those in the regional study (33.6%); in contrast, having more urgent or acute issues to address during consultations was less of a concern for physicians in Japan (36.7%; regional study: 83.6%). In both studies, physicians cited low patient willingness to vaccinate as a key barrier to recommending HZ vaccines.

### Limitations

No causal associations can be made from this self-administered, self-reported survey. Recall bias may have affected findings as responses could not be cross-checked or validated. For example, reporting bias could arise from differences in determining HZ awareness during screening (based on symptomatic description of HZ using local terms) versus during the survey (based on identifying HZ from a list of diseases). Specifically, the list of medical disease terms used in the survey could have contributed to cognitive overload and hence reporting bias, where a small subset of public respondents (older adults: 7.1%; adult children: 6.0%) who were aware of HZ as a disease were not aware of its medical term. Nevertheless, the screener confirming eligibility for survey participation ensured that all public respondents were aware of HZ as a disease.

Additionally, the study excluded respondents who are unaware of HZ or rejectors of preventative vaccines, as several interview/survey questions would not be relevant to them. The online survey format further excluded respondents who lack digital literacy. This purposive/selective sampling may thus have introduced selection bias, and study findings may not be representative of the overall Japanese population.

Finally, the physicians surveyed included only GPs, dermatologists, and pain clinicians, as these specialties are the most frequently involved in managing HZ cases. As KAP towards HZ and HZ vaccination may differ across specialties, study findings may not be representative of all physicians in Japan.

### Conclusion

This study reported the capability, opportunity, and motivation factors that could be addressed to overcome barriers hindering HZ vaccine uptake among patients aged ≥50 years in Japan. Overall, the public’s knowledge about HZ was low, and HZ vaccine affordability limited both vaccine recommendation by physicians and vaccine uptake among the public. Increased government-led education efforts to improve the knowledge of HZ, particularly its long-term complications, may encourage patient-physician discussions regarding HZ and support vaccine-seeking behavior given the influence of physician recommendation. Facilitating HZ vaccine access via providing and raising awareness of subsidies could further encourage joint patient-physician decision-making for HZ prevention.
