# Supplementary material for: Assessing Knowledge, Attitudes, and Practices Towards Herpes Zoster and Vaccination in Japan Using the Capability-Opportunity-Motivation-Behavior Model: a Mixed-Methods Study
Source: Int J Public Health. 2026 Mar 13;70:1608121. doi: 10.3389/ijph.2025.1608121 (PMC13021562; doi:10.3389/ijph.2025.1608121)
Supplement: Supplementary file 1 [file DataSheet1.docx]

# SUPPLEMENTARY MATERIALS

## Table S1. Key inclusion and exclusion criteria

| **Respondent group** | **Respondent subgroup** | **Inclusion criteria^a^** | **Exclusion criteria^b^** |
| --- | --- | --- | --- |
| Public respondents | HZ-naïve adults aged ≥50 years | 1. Aged ≥50 years 2. Aware of HZ 3. Open to preventive vaccination 4. Middle to upper income (for Phase 1) 5. HZ-naïve 6. Able to read and understand Japanese 7. Able to provide informed consent via email, and complete the virtual platform interview (for Phase 1) or online survey (for Phase 2) | Is a board-certified physician |
|  | Adults aged ≥50 years, vaccinated with ZVL or RZV | 1. Aged ≥50 years 2. Aware of HZ 3. Open to preventive vaccination 4. Middle to upper income (for Phase 1) 5. Vaccinated against HZ with ZVL or RZV 6. Able to read and understand Japanese 7. Able to provide informed consent via email, and complete the virtual platform interview (for Phase 1) or online survey (for Phase 2) |  |
|  | Current or former HZ patients aged ≥50 years | 1. Aged ≥50 years 2. Aware of HZ 3. Open to preventive vaccination 4. Middle to upper income (for Phase 1) 5. Currently experiencing or has been previously diagnosed with HZ 6. Able to read and understand Japanese 7. Able to provide informed consent via email, and complete the virtual platform interview (for Phase 1) or online survey (for Phase 2) |  |
|  | Working/financially independent adults aged 30–49 years, with parents aged ≥50 years | - 1. Aged 30–49 years   2. Has parent(s) aged ≥50 years   3. Aware of HZ   4. Key decision maker or makes decisions together as a family regarding healthcare (including vaccination) decisions for their parents   5. Open to preventive vaccination   6. Middle to upper income (for Phase 1)   7. Working or financially independent   8. Able to read and understand Japanese   9. Able to provide informed consent via email, and complete the virtual platform interview (for Phase 1) or online survey (for Phase 2) |  |
| Physician respondents | N/A | 1. Has at least 3 years of clinical experience in their specialty (GPs, pain clinicians, and dermatologists) 2. Treats HZ patients and/or is responsible for administering or recommending vaccines for adults in routine practice 3. Able to provide informed consent via email, and complete the virtual platform interview (for Phase 1) or online survey (for Phase 2) | Is a board-certified physician other than a GP, pain clinician, or dermatologist |

^a^Participants were required to meet the inclusion criteria to be eligible to enroll in Phases 1 and 2 of the study. ^b^Individuals meeting any of the exclusion criteria were not permitted to enroll in the study. **GP:** general practitioner; **HZ:** herpes zoster; **N/A:** not applicable; **RZV:** recombinant zoster vaccine; **ZVL:** zoster vaccine live.

## Table S2. Attitudes towards HZ, knowledge of long-term complications of HZ, and attitudes towards HZ vaccines among older adults aged ≥50 years, stratified by HZ vaccination status.

|  | **HZ-vaccinated (n=72)** | **Non-HZ-vaccinated (n=478)** | **p value** |
| --- | --- | --- | --- |
|  | **%** | **%** |  |
| **(A) Attitudes towards HZ** |  |  |  |
| Traditional chinese medicine (TCM) or other traditional medicine is my preferred treatment | 18.1 | 12.3 | 0.2980 |
| The older someone is the higher risk of contracting HZ | 90.3 | 86.0 | 0.4777 |
| The older someone is the higher risk of long-term complications from HZ | 87.5 | 75.5 | 0.0299** |
| If left untreated or treatment is delayed, HZ may lead to serious complications | 95.8 | 81.0 | 0.0073** |
| HZ has a negative effect on people's quality of life (e.g., social, emotional, and work) | 91.7 | 81.8 | 0.0411** |
| HZ can be costly to treat and manage | 59.7 | 46.9 | 0.0887 |
| It can be stressful for caregivers to care for patients with HZ | 76.4 | 62.8 | 0.0683 |
| HZ can affect people's ability to carry out activities of daily living | 90.3 | 85.8 | 0.5742 |
| One may die if the blister forms a complete circle/a ring around the body | 37.5 | 21.8 | 0.0089** |
| HZ can be treated with medication | 75.0 | 74.5 | 0.8074 |
| HZ can be prevented with vaccination | 97.2 | 86.6 | 0.0348** |
| **(B) Knowledge of long-term complications of HZ** | |  |  |
| Long-term nerve pain | 65.3 | 53.3 | 0.0579 |
| Loss of vision | 29.2 | 13.0 | 0.0003** |
| Loss of hearing | 19.4 | 9.6 | 0.0127** |
| Skin infection / scarring | 52.8 | 44.8 | 0.2036 |
| Insomnia | 12.5 | 8.8 | 0.3112 |
| Mood disorders like depression | 29.2 | 15.1 | 0.0029** |
| Facial nerve paralysis | 52.8 | 35.1 | 0.0040** |
| **(C) Attitudes towards HZ vaccines** |  |  |  |
| I’m worried about how the HZ vaccine may interact with treatments for my other conditions | 51.4 | 43.7 | 0.2098 |
| I’m worried about how the HZ vaccine may interact with other vaccines | 52.8 | 47.9 | 0.5762 |
| I’m worried about how the HZ vaccine may affect my other chronic conditions (e.g., diabetes, hypertension, high cholesterol) | 59.7 | 41.6 | 0.0142** |
| I prefer to acquire immunity naturally rather than get vaccinated | 26.4 | 35.4 | <0.0001*** |
| I am likely to get vaccinated if this is recommended by my doctor | 90.3 | 77.0 | 0.0365** |
| I want to get vaccinated but fear the side effects | 37.5 | 46.2 | 0.3566 |
| I want to get vaccinated to avoid the painful symptoms of HZ | 97.2 | 57.1 | <0.0001*** |
| I want to get vaccinated to avoid long term complications from HZ | 95.8 | 56.9 | <0.0001*** |
| I plan to speak to my doctor about the HZ vaccine | 54.2 | 24.9 | <0.0001*** |
| I leave it to my doctor to tell me if I should get the HZ vaccine | 41.7 | 35.1 | 0.0156** |
| My doctor did not recommend the HZ vaccine to me | 33.3 | 60.3 | <0.0001*** |
| It is convenient to get vaccinated for HZ | 80.6 | 52.5 | <0.0001*** |
| Not many people are getting the HZ vaccine | 51.4 | 49.6 | <0.0001*** |
| It is affordable | 47.2 | 31.8 | 0.0067** |
| I would like more information about the HZ vaccine from my doctor | 72.2 | 53.3 | 0.0106** |
| I rely on my friends/family for information on HZ vaccines | 16.7 | 9.6 | 0.0817 |

**p<0.05. ***p<0.0001. Survey questions for public respondents: **(A)** “Here are some other statements others have said about HZ. To what extent do you agree/disagree with these statements?” Data presented here are the proportions of respondents who indicated “Strongly agree” or “Agree” for each statement. **(B)** “What is/are the possible long-term complications* from HZ (shingles)? *By long-term complications we mean symptoms associated with HZ (shingles) for months and beyond. Please select all that apply.” Data presented here are the proportions of respondents who selected each option. **(C)** “The following are statements about what people think and feel about HZ (shingles) vaccine. To what extent do you agree/disagree with these statements?” Data presented here are the proportions of respondents who indicated “Strongly agree” or “Agree” for each statement. **HZ**: herpes zoster; **TCM**: traditional chinese medicine.

## Table S3. The public’s knowledge of risk factors, symptoms, and long-term complications of HZ

|  | **Older adults (N=550)** | | **Adult children (N=50)** | |
| --- | --- | --- | --- | --- |
|  | **n** | **%** | **n** | **%** |
| **(A) Knowledge of HZ risk factors** |  |  |  |  |
| Older age (aged ≥50 years) | 220 | 40.0 | 20 | 40.0 |
| Older age (aged ≥65 years) | 195 | 35.5 | 19 | 38.0 |
| Cancer | 20 | 3.6 | 5 | 10.0 |
| Chronic medical conditions (e.g., chronic obstructive pulmonary disease, diabetes mellitus, chronic/end-stage renal disease, asthma, and human immunodeficiency virus) | 50 | 9.1 | 8 | 16.0 |
| Treatment or medication for other diseases (e.g., cancer treatment) | 26 | 4.7 | 6 | 12.0 |
| Stress | 277 | 50.4 | 24 | 48.0 |
| Lack of sleep/insomnia | 155 | 28.2 | 18 | 36.0 |
| Previous chicken pox history | 163 | 29.6 | 12 | 24.0 |
| Previous measles history | 28 | 5.1 | 4 | 8.0 |
| Other infectious disease (e.g., COVID-19) | 32 | 5.8 | 11 | 22.0 |
| Low immunity/weakened immune system | 398 | 72.4 | 34 | 68.0 |
| Have poor personal hygiene | 37 | 6.7 | 6 | 12.0 |
| Unstable emotions/mental health disorders (e.g., depression, anxiety) | 42 | 7.6 | 8 | 16.0 |
| Poor diet/nutrition | 89 | 16.2 | 12 | 24.0 |
| Are in close contact with someone who has HZ | 74 | 13.5 | 18 | 36.0 |
| I do not know | 47 | 8.5 | 1 | 2.0 |
| **(B) Knowledge of HZ symptoms** |  |  |  |  |
| Rash | 411 | 74.7 | 38 | 76.0 |
| Pain | 455 | 82.7 | 37 | 74.0 |
| Itchiness & dryness | 232 | 42.2 | 22 | 44.0 |
| Skin numbness | 170 | 30.9 | 15 | 30.0 |
| Fever & chills | 143 | 26.0 | 17 | 34.0 |
| Headache | 44 | 8.0 | 6 | 12.0 |
| Upset stomach | 8 | 1.5 | 3 | 6.0 |
| Blisters that form a snake/band around the body | 415 | 75.5 | 26 | 52.0 |
| I do not know | 11 | 2.0 | 1 | 2.0 |
| **(C) Knowledge of HZ long-term complications** | |  |  |  |
| Long-term nerve pain | 302 | 54.9 | 25 | 50.0 |
| Loss of vision | 83 | 15.1 | 10 | 20.0 |
| Loss of hearing | 60 | 10.9 | 11 | 22.0 |
| Skin infection/scarring | 252 | 45.8 | 28 | 56.0 |
| Facial nerve paralysis (unable to control smiling, blinking and other facial movements) | 206 | 37.5 | 21 | 42.0 |
| Insomnia | 51 | 9.3 | 14 | 28.0 |
| Mood disorders like depression | 93 | 16.9 | 12 | 24.0 |
| Tingling | 1 | 0.2 | 0 | 0.0 |
| None – people get sick but recover fully | 19 | 3.5 | 1 | 2.0 |
| I do not know | 119 | 21.6 | 7 | 14.0 |

Survey questions for public respondents (data presented here are the proportions of respondents who selected each option): **(A)** “Which of the following are risk factors for developing or contracting HZ (shingles)? Please select all that apply.” **(B)** “Which of the following are symptoms are associated with HZ (shingles)? Please select all that apply.” **(C)** “What is/are the possible long-term complications* from HZ (shingles)? *By long-term complications we mean symptoms associated with HZ (shingles) for months and beyond. Please select all that apply.” COVID-19: coronavirus disease; **HZ**: herpes zoster.

## Table S4. Physician’s knowledge of the incidence rate, risk factors, and long-term complications of HZ

|  | **Physicians (N=60)** | |
| --- | --- | --- |
|  | **n** | **%** |
| **(A) Knowledge of HZ risk factors** |  |  |
| Aged ≥50 years | 47 | 78.3 |
| Cancer | 41 | 68.3 |
| Chronic medical conditions (e.g., chronic renal failure, diabetes mellitus, rheumatoid arthritis, and chronic pulmonary disease) | 46 | 76.7 |
| Human immunodeficiency virus | 40 | 66.7 |
| Other infectious disease (e.g., COVID-19) | 24 | 40.0 |
| Taking immunosuppressive medications | 51 | 85.0 |
| Bone marrow or solid organ transplant recipient | 36 | 60.0 |
| Inactive/sedentary lifestyle | 11 | 18.3 |
| Stressful lifestyle | 36 | 60.0 |
| None of the above | 0 | 0.0 |
| I do not know | 2 | 3.3 |
| **(B) Knowledge of HZ long-term complications** | |  |
| Post herpetic neuralgia | 58 | 96.7 |
| Loss of vision | 28 | 46.7 |
| Loss of hearing | 24 | 40.0 |
| Skin infection/scarring | 28 | 46.7 |
| Facial nerve paralysis | 38 | 63.3 |
| Insomnia | 23 | 38.3 |
| Mood disorders like depression | 25 | 41.7 |
| Others | 0 | 0.0 |
| None - people get sick but recover fully | 0 | 0.0 |
| **(C) Knowledge of HZ incidence rate** |  |  |
| 1 case per 1,000 person-years | 3 | 5.0 |
| 5 cases per 1,000 person-years | 15 | 25.0 |
| 10 cases per 1,000 person-years | 17 | 28.3 |
| 50 cases per 1,000 person-years | 17 | 28.3 |
| I do not know | 8 | 13.3 |

Survey questions for physician respondents (data presented here are the proportions of respondents who selected each option): **(A)** “Which of the following are risk factors for developing or contracting HZ? [Multiple responses are allowed]” **(B)** “The common possible long-term complications from HZ is/are… [Multiple responses are allowed]” **(C)** “Which of the following best approximates the incidence rate of HZ in the overall Japan population? [Please select a single response]” COVID-19: coronavirus disease; **HZ**: herpes zoster.

## Figure S1. Capability-Opportunity-Motivation-Behavior model for behavioral change


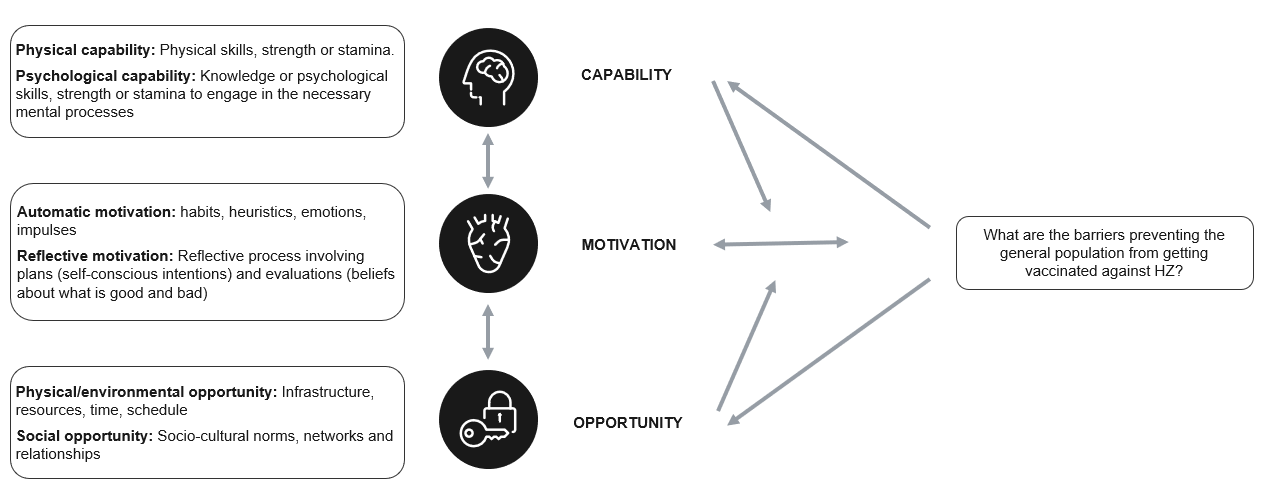


Reproduced from Chen J, et al. Hum Vaccin Immunother. 2024;20(1):2317446. doi: 10.1080/21645515.2024.2317446. **HZ:** herpes zoster.

## Figure S2. Study flowchart

Adapted from Chen J, et al. Hum Vaccin Immunother. 2024;20(1):2317446. doi: 10.1080/21645515.2024.2317446. ^a^The finalized questionnaire was based on a similar one developed and validated for the wider Asia-Pacific regional study, with adaption to the Japanese setting considering input from a local expert. **COM-B:** Capability-Opportunity-Motivation-Behavior; **IRB:** Institutional Review Board; **HZ:** herpes zoster; **KAP:** knowledge, attitudes, and practices.

## Figure S3. Phase 2 public respondents recruitment flow chart

**Eligible respondents meeting the inclusion criteria**

**Respondents who completed the questionnaire**

**N=600**

**Respondents invited to participate in the study**

**Exclusion criteria:**

- Respondents who are a board-certified physician

**Screened out:**

- Respondents who were unaware of HZ

N=1,566 (Japan)

- Respondents who reject preventive vaccines

N=2,033 (Japan)

**Respondents aged ≥50 years**

**N=550**

**Adult children (30-49 years old) with parents aged ≥50 years**

**N=50**

**HZ**: herpes zoster.

## Appendix S1. Phase 2 survey questionnaire for the public

**ZOASIS-J Extension**

Online survey to understand knowledge, attitudes and practices with regards to Herpes Zoster disease & vaccination

Questionnaire – General Population & Adult Children

| **Section** | **Questions covered** | **Outcome** |
| --- | --- | --- |
| **Section 1: General vaccine behavior** | - Awareness of vaccines - General knowledge of vaccines and what it does - What vaccines are important for them / parents and reasons? | **Evaluate awareness, knowledge, beliefs and perception towards general preventive vaccines** |
| **Section 2: Knowledge and beliefs about Herpes Zoster disease** | - Knowledge of symptoms and causes of Herpes Zoster, as well as risk factors, impact, recurrence, beliefs about how to prevent Herpes Zoster - Perception of impact of Herpes Zoster – physiological, psychological | **Evaluate knowledge, beliefs, habits, feelings around Herpes Zoster disease** |
| **Section 3: Herpes Zoster vaccines behavior**  **Knowledge, attitudes and practices towards Herpes Zoster vaccination** | - Awareness of Herpes Zoster vaccines and its role - Attitudinal statements – patient’s knowledge on Herpes Zoster / vaccine – including risk factors, impact, recurrence - Perception of impact of Herpes Zoster – physiological, psychological - Attitudinal statements – importance and benefits of Herpes Zoster vaccine | **Evaluate motivation and behavior around Herpes Zoster vaccination** |
| **Section 4 (Herpes Zoster patients only):**  **Experience and impact on HRQoL** | - Understand impact of Herpes Zoster disease and treatment-seeking behavior | **Understand impact of Herpes Zoster disease and treatment-seeking behavior** |
| **Section 5: Sources of information on Herpes Zoster and vaccine related information** | - Types of information desired on Herpes Zoster and Herpes Zoster vaccine - Key sources of information to obtain desired information vs what is trusted and preferred | **Assess unmet needs in terms of support desired** |

**Main Questionnaire**

**Section 1: General vaccine behavior**

1. Disease, vaccine awareness and status:
2. Which of the following disease/es are you aware of? Please select all that apply. [Multiple responses are allowed]

- Chicken Pox
- Diphtheria
- Hepatitis A
- Hepatitis B
- Influenza
- Measles
- Mumps
- Rubella
- Pertussis
- Tetanus
- Meningococcal disease
- Pneumococcal disease
- Herpes Zoster (Shingles) (Taijo-hoshin 帯状疱疹)
- HPV (Human Papillomavirus)
- None of the above

1. [Question is applicable to older adults aged 50 and above only]
   Which of the following have you personally experienced ***in the past 5 years***? Please select all that apply. [Multiple responses are allowed]

- Chicken Pox
- Diphtheria
- Hepatitis A
- Hepatitis B
- Influenza
- Measles
- Mumps
- Rubella
- Pertussis
- Tetanus
- Meningococcal disease
- Pneumococcal disease
- Herpes Zoster (Shingles) (Taijo-hoshin 帯状疱疹)
- HPV (Human Papillomavirus)
- None of the above

1. [Question is applicable to adult children only]
   Which of the following have your parents experienced ***in the past 5 years***? Please select all that apply. [Multiple responses are allowed]

- Chicken Pox
- Diphtheria
- Hepatitis A
- Hepatitis B
- Influenza
- Measles
- Mumps
- Rubella
- Pertussis
- Tetanus
- Meningococcal disease
- Pneumococcal disease
- Herpes Zoster (Shingles) (Taijo-hoshin 帯状疱疹)
- HPV (Human Papillomavirus)
- None of the above

1. Which of the following vaccine/s are you aware of? Please select all that apply. [Multiple responses are allowed]

- Chicken Pox
- Tetanus, diphtheria, and pertussis vaccine (Tdap)
- Hepatitis A
- Hepatitis B
- Influenza
- MMR (measles, mumps, and rubella) vaccine
- Meningococcal vaccine
- Pneumococcal vaccine
- Herpes Zoster (Shingles) (Taijo-hoshin 帯状疱疹)
- HPV (Human Papillomavirus)
- None of the above

1. [Question is applicable to older adults aged 50 and above only]
   Which of the following vaccine/s have you personally received ***in the past 5 years***? Please select all that apply. [Multiple responses are allowed]

- Chicken Pox
- Tetanus, diphtheria, and pertussis vaccine (Tdap)
- Hepatitis A
- Hepatitis B
- Influenza
- MMR (measles, mumps, and rubella) vaccine
- Meningococcal vaccine
- Pneumococcal vaccine
- Herpes Zoster (Shingles) (Taijo-hoshin 帯状疱疹)
- HPV (Human Papillomavirus)
- None of the above

1. [Question is applicable to adult children only]
   Which of the following vaccine/s have your elderly parents received ***in the past 5 years***? Please select all that apply. [Multiple responses are allowed]

- Chicken Pox
- Tetanus, diphtheria, and pertussis vaccine (Tdap)
- Hepatitis A
- Hepatitis B
- Influenza
- MMR (measles, mumps, and rubella) vaccine
- Meningococcal vaccine
- Pneumococcal vaccine
- Herpes Zoster (Shingles) (Taijo-hoshin 帯状疱疹)
- HPV (Human Papillomavirus)
- None of the above

1. Thinking of the **most recent vaccine that was NOT a COVID-19 vaccine…**
   1. [Question is applicable to older adults aged 50 and above only] that you personally received ***in the last 5 years***, what prompted you to get it?

Please select all that apply. [Multiple responses are allowed]

- Recommended by family / friend who does NOT have the disease
- Recommended by doctor / other Healthcare professionals
- No one recommended it. I / my parents heard/read about it
- No one recommended it. I / my parents habitually get vaccinated
- Requested / provided by my / my parent’s employer
- Recommended by family / friend who has the disease
- Other reasons (please specify): ________________
- Did not receive any adult vaccine other than COVID-19 vaccine
- Did not receive any adult vaccine including COVID-19 vaccine
  1. [Question is applicable to adult children only] that your elderly parent received, what prompted your parent to get it?

Please select all that apply. [Multiple responses are allowed]

- Recommended by family / friend who does NOT have the disease
- Recommended by doctor / other Healthcare professionals
- No one recommended it. I / my parents heard/read about it
- No one recommended it. I / my parents habitually get vaccinated
- Requested / provided by my / my parent’s employer
- Recommended by family / friend who has the disease
- Other reasons (please specify): ________________
- Did not receive any adult vaccine other than COVID-19 vaccine
- Did not receive any adult vaccine including COVID-19 vaccine

1. Which of the following factors are most important when deciding to get vaccinated for:
   1. [Question is applicable to older adults aged 50 and above only] You?

Please select the top 3 factors where “1” is most important and “2” is 2^nd^ most important and “3” is 3^rd^ most important. [Please select up to 3 responses]

**Values and Beliefs**

- Prevent getting the infection or getting the infection again
- Prevent long term complications of an illness
- Prevent severe illness
- Recommended by my family / friends
- Recommended by my doctor
- Recommended by other medical experts (example: infectious disease experts, etc.)
- Recommended by allied healthcare professionals (social workers, pharmacist, nurses)
- Recommended by the government
- Is affordable
- Convenient to get a vaccine
- Recommended by the media (example: TV, magazines, advertisements, social networking sites, etc.)
  1. [Question is applicable to adult children only] Your elderly parents?

Please select the top 3 factors where “1” is most important and “2” is 2^nd^ most important and “3” is 3^rd^ most important. [Please select up to 3 responses]

**Values and Beliefs:**

- Prevent getting the infection or getting the infection again
- Prevent long term complications of an illness
- Prevent severe illness
- Recommended by my family / friends
- Recommended by my doctor
- Recommended by other medical experts (example: infectious disease experts, etc.)
- Recommended by allied healthcare professionals (social workers, pharmacist, nurses)
- Recommended by the government
- Is affordable
- Convenient to get a vaccine
- Recommended by the media (example: TV, magazines, advertisements, social networking sites, etc.)

**Section 2****: Knowledge and beliefs about Herpes Zoster (Shingles) disease**

1. Which of the following are risk factors for developing or contracting Herpes Zoster (Shingles) (Taijo-hoshin 帯状疱疹)? Please select all that apply. [Multiple responses are allowed]

**Knowledge, beliefs**

- Older age (above 50 years)
- Older age (above 65 years)
- Cancer
- Chronic medical conditions (e.g., Chronic Obstructive Pulmonary Disease (COPD), diabetes mellitus, chronic / end-stage renal disease, asthma, human immunodeficiency virus (HIV))
- Treatment or medication for other diseases (e.g., cancer treatment)
- Stress
- Lack of sleep / insomnia
- Previous chicken pox history
- Previous measles history
- Other infectious disease (e.g., COVID-19)
- Low immunity / weakened immune system
- Have poor personal hygiene
- Unstable emotions / mental health disorders (e.g., depression, anxiety)
- Poor diet / nutrition
- Are in close contact with someone who has Herpes Zoster (Shingles) (Taijo-hoshin 帯状疱疹)
- Others (please specify): ________________
- I do not know

1. Which of the following are symptoms associated with Herpes Zoster (Shingles) (Taijo-hoshin 帯状疱疹)? Please select all that apply. [Multiple responses are allowed]

**Knowledge**

- Rash
- Pain
- Itchiness & dryness
- Skin numbness
- Fever & chills
- Headache
- Upset stomach
- Blisters that form a snake / band around the body
- I do not know

1. What is/are the possible long-term complications (sequelae)* from Herpes Zoster (Shingles) (Taijo-hoshin 帯状疱疹)? Please select all that apply. [Multiple responses are allowed]
   *By long-term complications (sequelae) we mean symptoms associated with Herpes Zoster (Shingles) (Taijo-hoshin 帯状疱疹) for months and beyond.

**Knowledge, Beliefs**

- Long-term nerve pain
- Loss of vision
- Loss of hearing
- Skin infection / scarring
- Facial nerve paralysis (unable to control smiling, blinking and other facial movements)
- Insomnia
- Mood disorders like depression
- Others (please specify): ________________
- None – people get sick but recover fully
- I do not know

1. Here are some statements others have said about Herpes Zoster (Shingles) (Taijo-hoshin 帯状疱疹) as an illness.

Please select a rating that is closest to how you feel about each statement.

[Please select a single response per statement]

To me, Herpes Zoster (Shingles) (Taijo-hoshin 帯状疱疹)...

| Is mild | 1 | 2 | 3 | 4 | 5 | Is severe | |
| --- | --- | --- | --- | --- | --- | --- | --- |
| Is uncommon | 1 | 2 | 3 | 4 | 5 | Is common | |
| Is curable | 1 | 2 | 3 | 4 | 5 | Is incurable | |
| Is preventable | 1 | 2 | 3 | 4 | 5 | Is not preventable | |
| A disease that one can only get once in a lifetime | 1 | 2 | 3 | 4 | 5 | May get it more than once | |
| [Applicable to older adults aged 50 and above only] | | | | | | |  |
| That I am at low risk of getting | 1 | 2 | 3 | 4 | 5 | That I am at high risk of getting | |
| [Applicable to adult children who responded that they are the primary decision maker for their parent / they make decisions together as a family only] | | | | | | |  |
| That my parents are at low risk of getting | 1 | 2 | 3 | 4 | 5 | That my parents are at high risk of getting | |
| A disease that one can make a full recovery with no complications (sequelae) | 1 | 2 | 3 | 4 | 5 | When infected, long term complications (sequelae) are possible | |
| Is not transmissible | 1 | 2 | 3 | 4 | 5 | Is highly transmissible | |

1. Here are some other statements others have said about Herpes Zoster (Shingles) (Taijo-hoshin 帯状疱疹).

To what extent do you agree/disagree with the statements? Please answer using the scale below. [Please select a single response per statement]

| TCM (Traditional Chinese Medicine) or other traditional medicine, is my preferred treatment | Strongly disagree | Disagree | Neutral | Agree | Strongly agree |
| --- | --- | --- | --- | --- | --- |
| The older someone is the higher risk of contracting Herpes Zoster (Shingles) (Taijo-hoshin 帯状疱疹) | Strongly disagree | Disagree | Neutral | Agree | Strongly agree |
| The older someone is the higher risk of long-term complications (sequelae) | Strongly disagree | Disagree | Neutral | Agree | Strongly agree |
| If left untreated or treatment is delayed, the disease may lead to serious complications (sequelae) | Strongly disagree | Disagree | Neutral | Agree | Strongly agree |
| It has a negative effect on people's quality of life (social, emotional, and work) | Strongly disagree | Disagree | Neutral | Agree | Strongly agree |
| It can be costly to treat and manage | Strongly disagree | Disagree | Neutral | Agree | Strongly agree |
| It can be stressful for caregivers to care for such patients | Strongly disagree | Disagree | Neutral | Agree | Strongly agree |
| It can affect people's ability to carry out activities of daily living (*activities of daily living could refer to toileting, getting dressed, eating, walking / climbing stairs, bathing / showering) | Strongly disagree | Disagree | Neutral | Agree | Strongly agree |
| One may die if the blister forms a complete circle / a ring around the body | Strongly disagree | Disagree | Neutral | Agree | Strongly agree |
| It can be treated with medication | Strongly disagree | Disagree | Neutral | Agree | Strongly agree |
| It can be prevented with vaccination | Strongly disagree | Disagree | Neutral | Agree | Strongly agree |

**Section 3: Herpes Zoster (Shingles) vaccines behavior**

1. Has anyone ever recommended the Herpes Zoster (Shingles) (Taijo-hoshin 帯状疱疹) vaccine
   1. [Question is applicable to older adults aged 50 and above only] To you? [Please select a single response]

- Yes
- No
  1. [Question is applicable to adult children only] To your elderly parents? [Please select a single response]
- Yes
- No
- I don’t know

1. If yes, who recommended it:
   1. [Question is applicable to older adults aged 50 and above who responded “yes” to Q9a] To you? Please select all that apply. [Multiple responses are allowed]

- Recommended by family / friend
- Recommended by doctor/ other Healthcare professionals
  1. [Question is applicable to adult children only who responded “yes” to Q9b] To your elderly parents? Please select all that apply. [Multiple responses are allowed]
- Recommended by family / friend
- Recommended by doctor/ other Healthcare professionals
- I don’t know

1. In your opinion, who should get vaccinated for Herpes Zoster (Shingles) (Taijo-hoshin 帯状疱疹)? Please select all that apply. [Multiple responses are allowed]

**Knowledge, Beliefs**

Those who….

- Are aged above 50
- Are aged above 65
- Have cancer
- Have Chronic medical conditions (e.g., Chronic Obstructive Pulmonary Disease (COPD), diabetes mellitus, chronic / end-stage renal disease, asthma, human immunodeficiency virus (HIV))
- Have insomnia / lack of sleep
- Had chicken pox before
- Had measles before
- Have low immunity / weakened immune system
- Have poor personal hygiene
- Have unstable emotions / mental health disorders (e.g., depression, anxiety)
- Have poor diet / nutrition
- Are in close contact with someone who has Herpes Zoster (Shingles) (Taijo-hoshin 帯状疱疹)
- Have too much sun exposure
- Lack exercise
- Had Herpes Zoster (Shingles) (Taijo-hoshin 帯状疱疹) before
- Have not been vaccinated with the Herpes Zoster (Shingles) (Taijo-hoshin 帯状疱疹) vaccine
- Have been previously vaccinated with the Herpes Zoster (Shingles) (Taijo-hoshin 帯状疱疹) vaccine
- I do not know

1. Do you plan to take the Herpes Zoster (Shingles) (Taijo-hoshin 帯状疱疹) vaccine in the future?
   1. [Question is applicable to older adults aged 50 and above who did not select ‘Herpes Zoster (Shingles) (Taijo-hoshin 帯状疱疹)’ for Q1e] For yourself? [Please select a single response]

- Yes
- No
- I don’t know / I have not decided
  1. [Question is applicable to adult children only who did not select ‘Herpes Zoster (Shingles) (Taijo-hoshin 帯状疱疹)’ for Q1f] For your elderly parents? [Please select a single response]
- Yes
- No
- I don’t know / I have not decided

1. Which of the following factors are most important when deciding whether to take the Herpes Zoster (Shingles) (Taijo-hoshin 帯状疱疹) vaccine:
   1. [Question is applicable to older adults aged 50 and above only] For yourself?
      Please select the top 3 factors where “1” is most important and “2” is 2^nd^ most important and “3” is 3^rd^ most important. [Please select up to 3 responses]

**Values and Beliefs**

- Prevent getting Herpes Zoster (Shingles) (Taijo-hoshin 帯状疱疹) disease (or getting it again)
- Prevent long term complications (sequelae) of Herpes Zoster (Shingles) (Taijo-hoshin 帯状疱疹)
- Prevent severe illness
- Recommended by my doctor
- Recommended by other medical expert (e.g., infectious disease experts, etc.)
- Recommended by allied healthcare professionals (e.g., social workers, pharmacist, nurses)
- Recommended by the government
- Recommended by family/friend
- My peers have received it
- Is affordable
- Is convenient to be vaccinated
- Is subsidized by my local government
  1. [Question is applicable to adult children only] For your elderly parents?
     Please select the top 3 factors where “1” is most important and “2” is 2nd most important and “3” is 3rd most important. [Please select up to 3 responses]

**Values and Beliefs**

- Prevent getting Herpes Zoster (Shingles) (Taijo-hoshin 帯状疱疹) disease (or getting it again)
- Prevent long term complications (sequelae) of Herpes Zoster (Shingles) (Taijo-hoshin 帯状疱疹)
- Prevent severe illness
- Recommended by my doctor
- Recommended by other medical expert (e.g., infectious disease experts, etc.)
- Recommended by allied healthcare professionals (e.g., social workers, pharmacist, nurses)
- Recommended by the government
- Recommended by family/friend
- My peers have received it
- Is affordable
- Is convenient to be vaccinated
- Is subsidized by my local government

14. The following are statements about what people think and feel about Herpes Zoster (Shingles) (Taijo-hoshin 帯状疱疹) vaccine.

To what extent do you agree with the following statements? Please answer using the below scale. [Please select a single response per statement]

| **Motivation** | | | | | |
| --- | --- | --- | --- | --- | --- |
| I’m worried about how the Herpes Zoster (Shingles) (Taijo-hoshin 帯状疱疹) vaccine may interact with treatments for my other conditions | Strongly disagree | Disagree | Neutral | Agree | Strongly agree |
| I’m worried about how the Herpes Zoster (Shingles) (Taijo-hoshin 帯状疱疹) vaccine may interact with other vaccines | Strongly disagree | Disagree | Neutral | Agree | Strongly agree |
| I’m worried about how the Herpes Zoster (Shingles) (Taijo-hoshin 帯状疱疹) vaccine may affect my other chronic conditions (e.g., Diabetes, hypertension, high cholesterol etc.) | Strongly disagree | Disagree | Neutral | Agree | Strongly agree |
| I prefer to acquire immunity naturally rather than get vaccinated | Strongly disagree | Disagree | Neutral | Agree | Strongly agree |
| I am likely to get vaccinated if this is recommended by my doctor | Strongly disagree | Disagree | Neutral | Agree | Strongly agree |
| I want to get vaccinated but fear the side effects | Strongly disagree | Disagree | Neutral | Agree | Strongly agree |
| I want to get vaccinated to avoid the painful symptoms of Herpes Zoster (Shingles) (Taijo-hoshin 帯状疱疹) | Strongly disagree | Disagree | Neutral | Agree | Strongly agree |
| I want to get vaccinated to avoid long term complications (sequelae) from Herpes Zoster (Shingles) (Taijo-hoshin 帯状疱疹) | Strongly disagree | Disagree | Neutral | Agree | Strongly agree |
| I plan to speak to my doctor about the Herpes Zoster (Shingles) (Taijo-hoshin 帯状疱疹) vaccination | Strongly disagree | Disagree | Neutral | Agree | Strongly agree |
| I leave it to my doctor to tell me if I should get the Herpes Zoster (Shingles) (Taijo-hoshin 帯状疱疹) vaccine | Strongly disagree | Disagree | Neutral | Agree | Strongly agree |
| **Physical and Social Opportunity** | | | | | |
| My doctor did not recommend the Herpes Zoster (Shingles) (Taijo-hoshin 帯状疱疹) vaccine to me | Strongly disagree | Disagree | Neutral | Agree | Strongly agree |
| It is convenient to get vaccinated for the Herpes Zoster (Shingles) (Taijo-hoshin 帯状疱疹) | Strongly disagree | Disagree | Neutral | Agree | Strongly agree |
| Not many people are getting the Herpes Zoster (Shingles) (Taijo-hoshin 帯状疱疹) vaccine | Strongly disagree | Disagree | Neutral | Agree | Strongly agree |
| It is affordable | Strongly disagree | Disagree | Neutral | Agree | Strongly agree |
| I would like more information about the Herpes Zoster (Shingles) (Taijo-hoshin 帯状疱疹) vaccine from my doctor | Strongly disagree | Disagree | Neutral | Agree | Strongly agree |
| I rely on my friends/family for information on Herpes Zoster (Taijo-hoshin 帯状疱疹) vaccines | Strongly disagree | Disagree | Neutral | Agree | Strongly agree |

**Section 4: Experience and impact on HRQoL**

[This section is applicable to Herpes Zoster **current / former patients** only]

1. You mentioned you had Herpes Zoster (Shingles) (Taijo-hoshin 帯状疱疹) previously. How did the illness affect you? [Please select a single response per statement]

| **HRQoL impact** | | | | | |
| --- | --- | --- | --- | --- | --- |
| Experienced pain during the illness | Not at all | A little bit | Some what | Quite a bit | Very much |
| Experienced pain that did not go away for months | Not at all | A little bit | Some what | Quite a bit | Very much |
| Experienced pain or other discomfort before rash appeared | Not at all | A little bit | Some what | Quite a bit | Very much |
| Impact to my emotions (e.g., depression) | Not at all | A little bit | Some what | Quite a bit | Very much |
| Unable to carry out activities of daily living (e.g., moving around, eating, taking a bath, grooming, going for a walk) | Not at all | A little bit | Some what | Quite a bit | Very much |
| Trouble sleeping/ sleeplessness | Not at all | A little bit | Some what | Quite a bit | Very much |
| Impact to physical health (e.g., loss of vision, hearing, headaches, itchiness and dryness) | Not at all | A little bit | Some what | Quite a bit | Very much |
| Unable to engage in hobbies (e.g., knitting, art/painting, cooking/baking, running) | Not at all | A little bit | Some what | Quite a bit | Very much |
| Unable to engage in social activities | Not at all | A little bit | Some what | Quite a bit | Very much |
| Unable to work | Not at all | A little bit | Some what | Quite a bit | Very much |
| Impact to learning, thinking, decision making or memory | Not at all | A little bit | Some what | Quite a bit | Very much |

1. When you last had Herpes Zoster (Shingles) (Taijo-hoshin 帯状疱疹), what treatment did you seek? Please select all that apply. [Multiple responses are allowed]

- Medical treatment (non-traditional medicine) at a clinic / hospital
- Over-the-counter (OTC) medication
- Traditional medicine (TCM or other traditional medicine)
- No treatment
- Others (please specify): ________________

1. [Question is applicable if ‘Medical treatment (non-traditional medicine) at a clinic / hospital’ is selected for Q16]
   Where did you seek medical treatment from? Please select all that apply. [Multiple responses are allowed]

- Primary care physicians (including general practitioner (GP)/family medicine (FM)/outpatient clinic in hospital/medical centres)
- Specialist treatment (dermatologist, infectious diseases (ID) physicians, pain specialist etc.)
- Emergency department

1. [Question is applicable if ‘Primary care physicians (including GP/FM/outpatient clinic in hospital/medical centres)’ is selected for Q17]
   When did you see a medical doctor? (by medical doctor, we **do not mean TCM (Traditional Chinese Medicine) or other traditional medication/treatment**)? [Please select a single response]

- Before the rash started
- Within 1-3 days when rash started
- Between 4-7 days when rash started
- More than a week but within 2 weeks after rash start
- More than 2 weeks after rash start

1. [Question is applicable if ‘Between 4-7 days when rash started’, ‘More than a week but within 2 weeks after rash start’, or ‘More than 2 weeks after rash start’ is selected for Q18]
   What was the reason for not seeking medical treatment earlier? Please select all that apply. [Multiple responses are allowed]

- I did not think the symptoms were severe
- I thought I could manage these symptoms on my own (with Over-the-counter or traditional medicine)
- I did not know which doctor to visit
- I could not afford treatment
- It was not convenient for me to seek medical treatment
- Others (please specify): ________________

**Section 5: Sources of information on Herpes Zoster (Shingles) and vaccine related information**

1. Regarding Herpes Zoster (Shingles) (Taijo-hoshin 帯状疱疹) disease, where or from whom have you heard about it? Please select all that apply. [Multiple responses are allowed]

- Healthcare professionals (e.g., Doctor/ nurses)
- My family member (s)
- My friends / peers
- Online support / community forum (Facebook groups, blogs, LINE)
- Independent health websites (Mayo clinic, WebMD)
- General internet searches
- Local media such as television, radio, newspapers
- People I follow on social media (online influencers, excluding family and friends)
- Social media (e.g., YouTube, Instagram, Twitter)
- Brochures / posters in hospitals / clinic
- A fellow patient advocate
- Websites of local government
- I have not heard of this from any of the above source

1. Regarding the availability of Herpes Zoster (Shingles) (Taijo-hoshin 帯状疱疹) vaccination, where or from whom have you heard about it? Please select all that apply. [Multiple responses are allowed]

- Healthcare professionals (e.g., Doctor/ nurses)
- My family member (s)
- My friends / peers
- Online support / community forum (Facebook groups, blogs, LINE)
- Independent health websites (Mayo clinic, WebMD)
- General internet searches
- Local media such as television, radio, newspapers
- People I follow on social media (online influencers, excluding family and friends)
- Social media (e.g., YouTube, Instagram, Twitter)
- Brochures / posters in hospitals / clinic
- A fellow patient advocate
- Websites of local government
- I have not heard of this from any of the above source

1. Which channels / source would you trust the most? Please rank the top 3.

[Please select up to 3 responses]

- Healthcare professionals (e.g., Doctor/ nurses)
- My family member (s)
- My friends / peers
- Online support / community forum (Facebook groups, blogs, LINE)
- Independent health websites (Mayo clinic, WebMD)
- General internet searches
- Local media such as television, radio, newspapers
- People I follow on social media (online influencers, excluding family and friends)
- Social media (e.g., YouTube, Instagram, Twitter)
- Brochures / posters in hospitals / clinic
- A fellow patient advocate
- Websites of local government

1. Which channels / source do you prefer to receive information from? Please rank the top 3.

[Please select up to 3 responses]

- Healthcare professionals (e.g., Doctor/ nurses)
- My family member (s)
- My friends / peers
- Online support / community forum (Facebook groups, blogs, LINE)
- Independent health websites (Mayo clinic, WebMD)
- General internet searches
- Local media such as television, radio, newspapers
- People I follow on social media (online influencers, excluding family and friends)
- Social media (e.g., YouTube, Instagram, Twitter)
- Brochures / posters in hospitals / clinic
- A fellow patient advocate
- Websites of local government

1. ***In the past year***, have you seen any news or information being provided in the media (newspapers, radio, TV, internet etc) about Herpes Zoster (Shingles) (Taijo-hoshin 帯状疱疹) disease and how to prevent it? [Please select a single response]

- Yes
- No

1. What topics would you like to be provided with to help you learn more about Herpes Zoster (Shingles) (Taijo-hoshin 帯状疱疹) disease and vaccine? Please select all that apply.

[Multiple responses are allowed]

- Information about symptoms of Herpes Zoster (Shingles) (Taijo-hoshin 帯状疱疹)
- Information about causes of Herpes Zoster (Shingles) (Taijo-hoshin 帯状疱疹)
- Information about complications (sequelae) of Herpes Zoster (Shingles) (Taijo-hoshin 帯状疱疹)
- Information about risk factors for Herpes Zoster (Shingles) (Taijo-hoshin 帯状疱疹)
- Information about vaccine safety
- Local recommendation on the vaccine
- Who should be vaccinated against Herpes Zoster (Shingles) (Taijo-hoshin 帯状疱疹)
- When to get vaccinated for Herpes Zoster (Shingles) (Taijo-hoshin 帯状疱疹)
- Where to get vaccinated for Herpes Zoster (Shingles) (Taijo-hoshin 帯状疱疹)
- Cost of Herpes Zoster (Shingles) (Taijo-hoshin 帯状疱疹) vaccine
- Experience shared by others who have received the vaccination
- Experiences shared by ex / current Herpes Zoster (Taijo-hoshin 帯状疱疹) patients
- Where the vaccine is manufactured
- How the vaccine is manufactured
- Number of injections needed (e.g., one dose or two doses)
- Number of people who have received the vaccine around the world
- Number of people who have received the vaccine in this country
- Known side effects and risks from the vaccine
- Effectiveness of the vaccine
- Where the vaccine is approved
- Others (please specify): ________________

**End of questionnaire**

## Appendix S2. Phase 2 survey questionnaire for physicians

**ZOASIS-J Extension**

Online survey to understand knowledge, attitudes and practices with regards to Herpes Zoster disease & vaccination

Questionnaire – Healthcare Professional

| **Section** | **Questions covered** | **Outcome** |
| --- | --- | --- |
| **Section 1: HCP’s general vaccine behavior** | - General vaccine behavior - Disease, vaccine awareness and status of patients - What motivated the patient to get vaccinated? - Proportion of patients they have initiated a conversation with around vaccines - Factors that are important when considering a vaccine (from patient’s perspective) | **Evaluate awareness, knowledge, and perception towards general preventive vaccines** |
| **Section 2: Knowledge and attitudes about Herpes Zoster** | - Knowledge around incidence, risk factors and long-term complications of Herpes Zoster - Attitudinal statements about Herpes Zoster | **Understand motivations around Herpes Zoster disease and vaccine behavior** |
| **Section 3: Knowledge and attitudes about Herpes Zoster vaccine** | - Likelihood to recommend a vaccine - Attitudinal statements about vaccines |  |
| **Section 4: HCPs’ experience with Herpes Zoster** | - Proportion of patients recommended with Herpes Zoster vaccine - Proportion of patients likely to initiate a vaccine conversation with - Proportion of patients to seek medical care – from onset of rash | **Evaluate practice towards Herpes Zoster vaccines and experience with Herpes Zoster** |

**Main Questionnaire**

**Section 1: HCP’s general vaccine behavior**

1. Which of the following statement is applicable in your current practice? [Multiple responses are allowed]

- I recommend vaccines to some of my patients aged ≥50 years
- I prescribe vaccines to some of my patients aged ≥50 years
- I administer vaccines to some of my patients aged ≥50 years
- None of the above

1. Thinking of YOUR adult patients **(18 years and above)**:
   1. Which of the following vaccine/s do you recommend in the past 5 years? [Multiple responses are allowed]

**Disease, vaccine awareness and status**

- Hepatitis A vaccine (HepA)
- Hepatitis B vaccine (HepB)
- Herpes Zoster vaccine
- HPV (Human papillomavirus) vaccine
- Influenza vaccine
- Measles, mumps, and rubella vaccine (MMR)
- Meningococcal vaccine (MenACWY or MenB)
- Pneumococcal vaccine (PPSV23 or PCV13)
- Tetanus diphtheria toxoids (Td)
- Tetanus, diphtheria, and pertussis vaccine (Tdap)
- Varicella vaccine (VAR)
- Others (please specify): ________________
- None of the above
  1. Which of the following vaccine/s do patients ask for without you recommending in the past 5 years? [Multiple responses are allowed]

**Disease, vaccine awareness and status**

- Hepatitis A vaccine (HepA)
- Hepatitis B vaccine (HepB)
- Herpes Zoster vaccine
- HPV (Human papillomavirus) vaccine
- Influenza vaccine
- Measles, mumps, and rubella vaccine (MMR)
- Meningococcal vaccine (MenACWY or MenB)
- Pneumococcal vaccine (PPSV23 or PCV13)
- Tetanus diphtheria toxoids (Td)
- Tetanus, diphtheria, and pertussis vaccine (Tdap)
- Varicella vaccine (VAR)
- Others (please specify): ________________
- None of the above

1. Thinking of YOUR **patients aged ≥50 years old**,
2. Which vaccination/s is/are important to recommend? [Multiple responses are allowed]

- Hepatitis A vaccine (HepA)
- Hepatitis B vaccine (HepB)
- Herpes Zoster vaccine
- Influenza vaccine
- Measles, mumps, and rubella vaccine (MMR)
- Meningococcal vaccine (MenACWY or MenB)
- Pneumococcal vaccine (PPSV23 or PCV13)
- Tetanus diphtheria toxoids (Td)
- Tetanus, diphtheria, and pertussis vaccine (Tdap)
- Varicella vaccine (VAR)
- None of the above

1. Over the **past 6 months**, which of the following vaccines have you recommended, prescribed and / or administered to any patients aged ≥50 years? [Multiple responses are allowed]

- Hepatitis A vaccine (HepA)
- Hepatitis B vaccine (HepB)
- Herpes Zoster vaccine
- Influenza vaccine
- Measles, mumps, and rubella vaccine (MMR)
- Meningococcal vaccine (MenACWY or MenB)
- Pneumococcal vaccine (PPSV23 or PCV13)
- Tetanus diphtheria toxoids (Td)
- Tetanus, diphtheria, and pertussis vaccine (Tdap)
- Varicella vaccine (VAR)
- None of the above

1. Over the **past year**, what proportion of your **patients aged ≥50 years** did you initiate a conversation about any vaccination (other than COVID-19 vaccine)?

_________% [Please enter a whole number between 0 to 100 only]

1. For **patients aged ≥50 years**, which of the following factors are important when you decide which vaccines to prioritize recommendation for? Please select the top 3 factors where “1” is most important” and “2” is 2^nd^ most important and “3” is 3^rd^ most important. [Please select up to 3 responses]

- Disease prevalence (frequency and risk of occurrence)
- Impact of disease to health conditions and complications
- Underlying disease of patient
- Patients’ views on whether the vaccine is a priority
- Type of vaccine (Live attenuated, inactivated, recombinant, mRNA, etc.)
- Age of patients
- Cost of vaccine (including subsidy provided)
- Storage space in my hospital / clinic
- Dosing is convenient
- Recommended by government / national vaccination list
- Recommended by international guidelines
- Recommended by national professional societies (Academic Society)

**Section 2: Knowledge and attitudes about Herpes Zoster**

Based on your personal real-world clinical experience about Herpes Zoster and patients, please answer the following questions:

1. Which of the following best approximates the incidence rate of Herpes Zoster in the overall Japan population? [Please select a single response]

**Knowledge, Beliefs**

- 1 case per 1,000 person-years
- 5 cases per 1,000 person-years
- 10 cases per 1,000 person-years
- 50 cases per 1,000 person-years
- I do not know

1. Which of the following are risk factors for developing or contracting Herpes Zoster? [Multiple responses are allowed]

**Knowledge, Beliefs**

- Aged 50 years or older
- Cancer
- Chronic medical conditions (e.g., chronic renal failure, diabetes mellitus, rheumatoid arthritis, and chronic pulmonary disease)
- Human immunodeficiency virus (HIV)
- Other infectious disease (e.g., COVID-19)
- Taking immunosuppressive medications
- Bone marrow or solid organ transplant recipient
- Inactive / sedentary lifestyle
- Stressful lifestyle
- None of the above
- I do not know

1. The common possible long-term complications (sequelae) from Herpes Zoster is/are…

[Multiple responses are allowed]

**Knowledge, Beliefs**

- Post herpetic neuralgia
- Loss of vision
- Loss of hearing
- Skin infection / scarring
- Facial nerve paralysis
- Insomnia
- Mood disorders like depression
- Others (please specify): ________________
- None – people get sick but recover fully

1. Here are some statements that other clinicians have told us are important to know about Herpes Zoster disease.

Please rate the importance for you personally, using the scale below.

[Please select a single response per statement]

**Capability**
Having knowledge about...

| The clinical presentation of Herpes Zoster | Not at all important | A bit important | Neutral | Quite important | Very important |
| --- | --- | --- | --- | --- | --- |
| Herpes Zoster disease management | Not at all important | A bit important | Neutral | Quite important | Very important |
| Long-term effects and complications of Herpes Zoster | Not at all important | A bit important | Neutral | Quite important | Very important |
| Risk factors for Herpes Zoster | Not at all important | A bit important | Neutral | Quite important | Very important |
| Impact of Herpes Zoster on chronic diseases such as diabetes, COPD, etc. | Not at all important | A bit important | Neutral | Quite important | Very important |
| Prevention methods of Herpes Zoster | Not at all important | A bit important | Neutral | Quite important | Very important |

1. Here are some statements about Herpes Zoster disease. To what extent do you agree/disagree with the statements?

Please answer using the scale below. [Please select a single response per statement]

| **Motivation** | | | | | |
| --- | --- | --- | --- | --- | --- |
| Herpes Zoster can have a negative effect on people's quality of life (social, emotional, and work) | Strongly disagree | Disagree | Neutral | Agree | Strongly Agree |
| It can be costly to treat and manage Herpes Zoster | Strongly disagree | Disagree | Neutral | Agree | Strongly Agree |
| It can be stressful for caregivers to care for Herpes Zoster patients | Strongly disagree | Disagree | Neutral | Agree | Strongly Agree |
| It can affect people's ability to carry out activities of daily living | Strongly disagree | Disagree | Neutral | Agree | Strongly Agree |
| Herpes Zoster is a serious risk to patients with underlying chronic conditions | Strongly disagree | Disagree | Neutral | Agree | Strongly Agree |
| **Opportunity** | | | | | |
| It is important to educate the general population about the Herpes Zoster disease | Strongly disagree | Disagree | Neutral | Agree | Strongly Agree |
| My peers and I share information and updates about the Herpes Zoster disease | Strongly disagree | Disagree | Neutral | Agree | Strongly Agree |
| It is important to educate the general population about the re-occurrence of Herpes Zoster | Strongly disagree | Disagree | Neutral | Agree | Strongly Agree |
| It is important to educate the general population to seek early treatment when presenting with symptoms of Herpes Zoster | Strongly disagree | Disagree | Neutral | Agree | Strongly Agree |

**Section 3: Knowledge and attitudes about Herpes Zoster Vaccine**

1. What factors do you consider before recommending Herpes Zoster vaccine to **adult patients aged ≥50 years**? [Multiple responses are allowed]

**Knowledge, Beliefs**

- Age
- Level of immunosuppression or severity of underlying diseases
- Willingness / motivation of the patient to be vaccinated
- Medical history (including co-morbidities)
- Vaccination history (documented or patient reported from memory)
- Patient's financial ability
- Patient's ability to understand the disease
- Season / time of the year (e.g. adult vaccination cycle). Please specify specific season / time of the year: ________________
- Others (please specify): ________________

1. Under what circumstances do you **NOT** initiate conversations with … regarding Herpes Zoster vaccination? [Multiple responses are allowed]
   1. [Question is applicable to adult patients aged ≥50 years only]
   - There is not enough time
   - There are more urgent or acute issues to discuss with patients during the consultation
   - The patient has already been vaccinated for Herpes Zoster
   - I think the risk of Herpes Zoster is low in this patient population
   - Other healthcare providers (e.g. GPs / other specialists) have already discussed Herpes Zoster vaccination with this patient population
   - I am uncertain about what the local medical society or other guidelines say about vaccinating this population for the prevention of Herpes Zoster
   - Some of my patients cannot afford to pay for the vaccine
   - Willingness/motivation of patients to be vaccinated is low
   - Patient's ability to understand the disease is low
   - Others (please specify): ________________
   1. [Question is applicable to adult patients aged ≥50 years with chronic conditions such as diabetes, COPD, etc. only]
   - There is not enough time
   - There are more urgent or acute issues to discuss with patients during the consultation
   - The patient has already been vaccinated for Herpes Zoster
   - I think the risk of Herpes Zoster is low in this patient population
   - Other healthcare providers (e.g. GPs / other specialists) have already discussed Herpes Zoster vaccination with this patient population
   - I am uncertain about what the local medical society or other guidelines say about vaccinating this population for the prevention of Herpes Zoster
   - Some of my patients cannot afford to pay for the vaccine
   - Willingness/motivation of patients to be vaccinated is low
   - Patient's ability to understand the disease is low
   - Others (please specify): ________________
2. Here are some statements about Herpes Zoster vaccine. Please rate the importance for you personally, using the scale below. [Please select a single response per statement]

| Patients aged ≥50 years should get vaccinated for Herpes Zoster | Not at all important | A bit important | Somewhat important | Quite important | Very important |
| --- | --- | --- | --- | --- | --- |
| Patients ≥50 years with chronic conditions such as diabetes, COPD etc. should get vaccinated for Herpes Zoster | Not at all important | A bit important | Somewhat important | Quite important | Very important |
| Patients with immunocompromised diseases / undergoing immunosuppressive treatment should be vaccinated | Not at all important | A bit important | Somewhat important | Quite important | Very important |

1. How likely are you to recommend a Herpes Zoster vaccine to….? [Please select a single response per statement]

| Patients aged ≥50 years | Extremely unlikely | Very unlikely | Somewhat likely | Very likely | Extremely likely |
| --- | --- | --- | --- | --- | --- |
| Patients ≥50 years with chronic conditions such as diabetes, COPD etc. | Extremely unlikely | Very unlikely | Somewhat likely | Very likely | Extremely likely |
| Patients with immunocompromised diseases / undergoing immunosuppressive treatment should be vaccinated | Extremely unlikely | Very unlikely | Somewhat likely | Very likely | Extremely likely |

1. Here are some statements that other clinicians have told us are important to know about Herpes Zoster vaccine. Please rate the importance for you personally, using the scale below. [Please select a single response per statement]

**Capability**

Having knowledge about...

| Which patient population I should recommend the Herpes Zoster vaccine to | Not at all important | A bit important | Neutral | Quite important | Very important |
| --- | --- | --- | --- | --- | --- |
| The latest Herpes Zoster vaccine scientific data | Not at all important | A bit important | Neutral | Quite important | Very important |
| Side effects of the Herpes Zoster vaccines | Not at all important | A bit important | Neutral | Quite important | Very important |
| Herpes Zoster vaccines’ contraindications | Not at all important | A bit important | Neutral | Quite important | Very important |
| Which Herpes Zoster vaccine options are available in my market | Not at all important | A bit important | Neutral | Quite important | Very important |
| Advantages and disadvantages of the different Herpes Zoster vaccines | Not at all important | A bit important | Neutral | Quite important | Very important |
| National guidelines and recommendations for the Herpes Zoster vaccines | Not at all important | A bit important | Neutral | Quite important | Very important |
| Recommendations from national professional societies | Not at all important | A bit important | Neutral | Quite important | Very important |
| Medical subsidies and reimbursements available (or lack of) in my market for Herpes Zoster vaccines | Not at all important | A bit important | Neutral | Quite important | Very important |
| Age at which Herpes Zoster vaccination is indicated | Not at all important | A bit important | Neutral | Quite important | Very important |

16. Here are some statements about Herpes Zoster vaccination.

To what extent do you agree/ disagree with the statements? Please answer using the scale below: [Please select a single response per statement]

| **Motivation** | | | | | |
| --- | --- | --- | --- | --- | --- |
| It is important for people to get vaccinated to reduce the risk of long-term complications (sequelae) from Herpes Zoster | Strongly disagree | Disagree | Neutral | Agree | Strongly agree |
| Patients having certain underlying conditions would make me prioritize the Herpes Zoster vaccine | Strongly disagree | Disagree | Neutral | Agree | Strongly agree |
| Recommending Herpes Zoster vaccination is in line with my clinical goals | Strongly disagree | Disagree | Neutral | Agree | Strongly agree |
| I will only recommend Herpes Zoster vaccination to patients I have a good relationship and rapport with | Strongly disagree | Disagree | Neutral | Agree | Strongly agree |
| I find it easy to talk to my patients about their health and vaccinations | Strongly disagree | Disagree | Neutral | Agree | Strongly agree |
| I feel comfortable recommending Herpes Zoster vaccination to my patients | Strongly disagree | Disagree | Neutral | Agree | Strongly agree |
| Patients usually accept my vaccination recommendations | Strongly disagree | Disagree | Neutral | Agree | Strongly agree |
| There is a need to recommend Herpes Zoster vaccination to former Herpes Zoster patients to reduce the risk of recurrence | Strongly disagree | Disagree | Neutral | Agree | Strongly agree |
| **Opportunity** | | | | | |
| It is important to educate the general population about Herpes Zoster vaccination | Strongly disagree | Disagree | Neutral | Agree | Strongly agree |
| My peers and I share information and updates about the Herpes Zoster vaccine | Strongly disagree | Disagree | Neutral | Agree | Strongly agree |
| My peers encourage their patients to receive Herpes Zoster vaccination | Strongly disagree | Disagree | Neutral | Agree | Strongly agree |
| Official government information, guidelines or campaigns influence my Herpes Zoster vaccine recommendation | Strongly disagree | Disagree | Neutral | Agree | Strongly agree |
| It is convenient to store the Herpes Zoster vaccine in my clinic | Strongly disagree | Disagree | Neutral | Agree | Strongly agree |
| I have time to speak to my patients about Herpes Zoster and Herpes Zoster vaccination | Strongly disagree | Disagree | Neutral | Agree | Strongly agree |
| It is easy for me to identify patients who need Herpes Zoster vaccination | Strongly disagree | Disagree | Neutral | Agree | Strongly agree |
| There is enough communication in the general media about Herpes Zoster and Herpes Zoster vaccination | Strongly disagree | Disagree | Neutral | Agree | Strongly agree |

**Section 4: HCPs’ experience with Herpes Zoster**

1. Over the past year, approximately…
   1. How many cases of Herpes Zoster patients aged ≥50 years old have you personally encountered in your practice? [Please enter numeric figures only – Minimum 0, Maximum 2000]

| ___________________________________ | Herpes Zoster patients aged ≥50 years old |
| --- | --- |

- 1. How many cases of Herpes Zoster patients aged ≥50 years old presenting with chronic conditions such as diabetes, COPD, etc. have you personally encountered in your practice? [Please enter numeric figures only – Minimum 0, Maximum 2000]

| ___________________________________ | Herpes Zoster patients aged ≥50 years old presenting with chronic conditions (diabetes, COPD, etc…) |
| --- | --- |

18. Thinking back in the **past year** of the Herpes Zoster patients you have personally managed…

From their initial onset of symptoms, when did your patients first seek medical care (medical care does not include Traditional Chinese Medicine treatment)? [Please enter numeric figures only – all numbers should sum up to 100%]

| **Description** | **% of patients** |
| --- | --- |
| Before the rash started |  |
| Within 1-3 days when rash started |  |
| Between 4-7 days when rash started |  |
| More than a week but within 2 weeks after rash start |  |
| More than 2 weeks after rash start |  |
| Total | 100% |

1. Over the **past year**, approximately…
2. What proportion of your **patients aged ≥50 years** did you initiate a conversation about Herpes Zoster vaccination? [Please enter a whole number between 0 to 100 only]

| ____________________________% | patients aged ≥50 years old |
| --- | --- |

1. What proportion of your **patients aged ≥50 years presenting with chronic conditions** such as diabetes, COPD, etc. did you initiate a conversation about Herpes Zoster vaccination? [Please enter a whole number between 0 to 100 only]

| ____________________________% | patients aged ≥50 years old presenting with chronic conditions (diabetes, COPD, etc.) |
| --- | --- |

**End of questionnaire**
